# Preclinical characterization of FAP-targeting radioconjugates: assessing biophysical and pharmacokinetic equivalence of lutetium-177 and terbium-161

**DOI:** 10.1186/s13550-026-01488-8

**Published:** 2026-07-29

**Authors:** Ana Katrina Mapanao, Ecem Selin Adakli, Christian Vaccarin, Fan Sozzi-Guo, Anzhelika N. Moiseeva, Roger Schibli, Nicholas P. van der Meulen, Cristina Müller, Thera4Care Consortium

**Affiliations:** 1 Center for Radiopharmaceutical Sciences, PSI Center for Life Sciences, Villigen‑PSI, Switzerland; 2 Institute of Pharmaceutical Sciences, Department of Chemistry and Applied Biosciences, ETH Zurich, Zurich, Switzerland; 3 Laboratory of Radiochemistry, PSI Center for Nuclear Engineering and Sciences, Villigen‑PSI, Switzerland

**Keywords:** Fibroblast activation protein, FAP inhibitors, Terbium-161, Lutetium-177, Fibrosarcoma, Radionuclide therapy

## Abstract

**Background:**

Radioconjugates targeting the fibroblast activation protein (FAP) have been successfully applied for nuclear imaging and are currently under investigation for radionuclide therapy. The aim of this study was to assess the biophysical properties of the ^177^Lu-labeled FAP inhibitors (FAPIs), FAPI-46, OncoFAP, FAP-2286 and 3BP-3940, and compare them with their ^161^Tb-labeled analogues. The respective ^177^Lu- and ^161^Tb-labeled FAPIs were evaluated using fibrosarcoma tumor cells in vitro and a xenograft mouse model to demonstrate pharmacokinetic equivalence.

**Results:**

The radioconjugates (500 MBq/mL) remained ≥ 96% intact in saline over 24 h, irrespective of the employed radionuclide. The ^161^Tb-labeled conjugates showed similar logD values, uptake in HT1080-FAP cells and FAP-binding affinities (K_D_: 0.48–1.8 nM) as their ^177^Lu-based analogues. The in vitro binding to mouse and human blood plasma proteins was moderate with protein-bound fractions in the ranges of 39–56% and 31–61%, respectively, irrespective of the employed radionuclide. After 24 h incubation, the intact fraction of radioconjugates in mouse blood plasma was ≥ 87% while this was even ≥ 91% in human blood plasma without any differences among the same FAPIs labeled with either radionuclide. In HT1080-FAP/HT1080 tumor-bearing mice, the ^161^Tb-based FAP-targeting radioconjugates showed distribution profiles that corresponded well with their ^177^Lu-labeled analogues as demonstrated by dual-isotope SPECT/CT imaging and quantitative biodistribution data obtained at 4 h post-injection (p.i.). The radioconjugates were rapidly cleared from the blood, with < 1.5% IA/g retained at 1 h after administration. HT1080-FAP tumor accumulation reached its maximum with 8.8–20% IA/g at 1 h after injection of the radioconjugates, followed by gradual wash-out to 7.4–11% IA/g at 4 h p.i. and further to < 3% IA/g at 24 h p.i. An exception was the 3BP-3940 radioconjugate which still showed 6.4 ± 0.6% IA/g in the tumor xenograft at 24 h p.i..

**Conclusion:**

The in vitro and in vivo properties of the ^161^Tb-labeled radioconjugates were similar to those of the ^177^Lu-labeled analogues. The accumulation of these FAP-targeting radioconjugates in HT1080-FAP tumors of mice was reasonably high, however, as the activity declined rapidly over time, repeated radioconjugate administration may be necessary to achieve therapeutic efficacy.

**Graphical abstract:**

**Figure:**
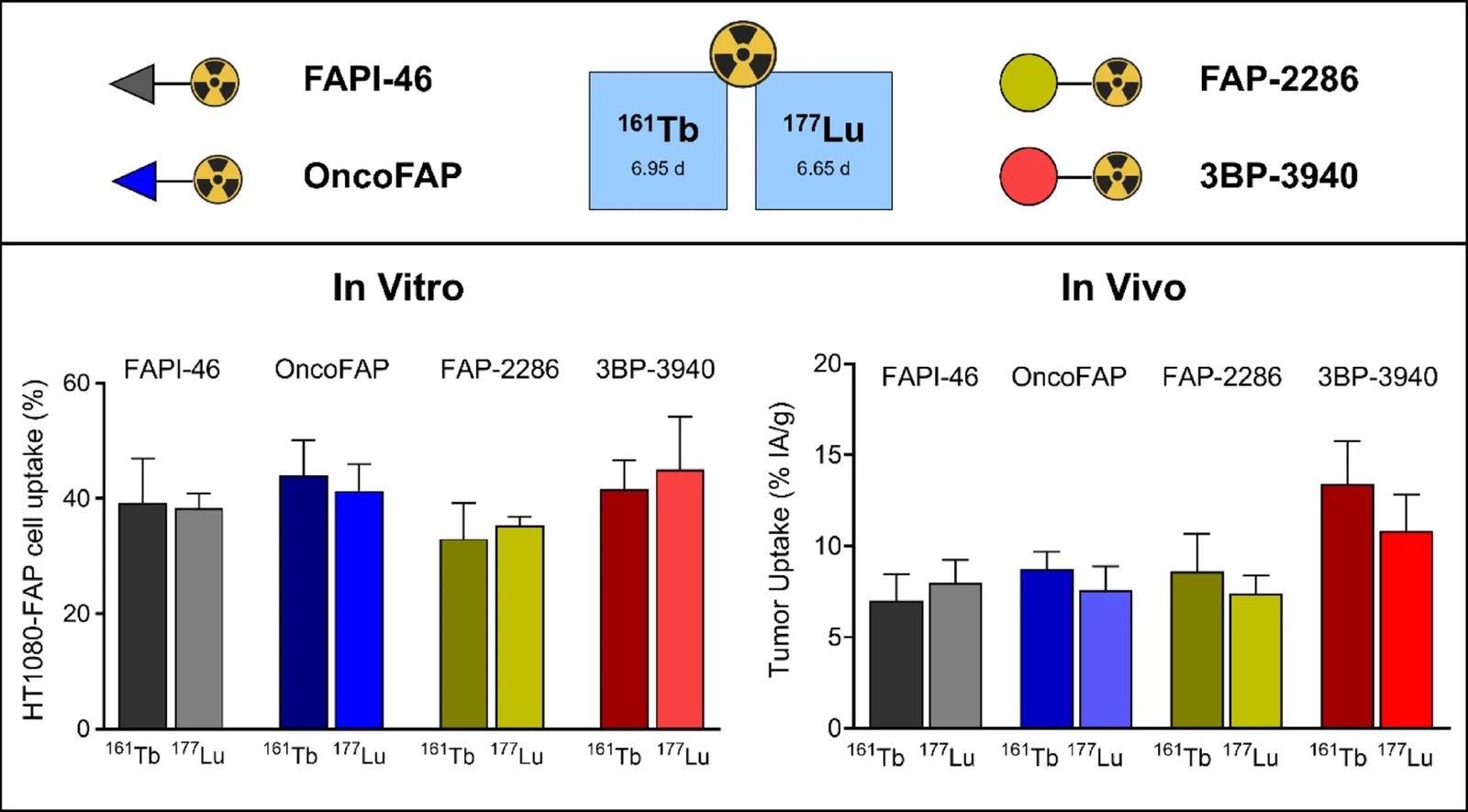

**Supplementary Information:**

The online version contains supplementary material available at https://doi.org/10.1186/s13550-026-01488-8.

## Introduction

The fibroblast activation protein (FAP), a type II transmembrane serine protease, is mostly expressed on cancer-associated fibroblasts (CAFs) of the tumor microenvironment [1], which makes it an interesting target for nuclear imaging of a multitude of cancers [2]. Initially, UAMC-1110, a small molecular weight FAP inhibitor (FAPI), served as the basis for the radioconjugate design [3]. Among these, [^68^Ga]Ga-FAPI-04 and [^68^Ga]Ga-FAPI-46 showed high uptake in diverse tumor types and favorable tumor-to-background ratios on positron emission tomography (PET) images [4]. The feasibility of using FAP for CAF-targeted radionuclide therapy is still not clear and preclinical investigations will be necessary to assess the concept of targeting the microenvironment with therapeutic radiopharmaceuticals [5].

In sarcoma, a rare malignant tumor type that originates in the connective tissues such as bone, fat, muscle or nerves [6], FAP was found to be expressed on tumor cells in addition to CAFs [7, 8]. High expression was found in patients with advanced stage osteosarcoma, which was associated with shorter overall survival as compared to FAP-negative cases [9]. In the case of sarcoma, FAP may, thus, not only be an interesting target for diagnostic imaging but also for targeted radionuclide therapy as it would follow the same concept of targeting tumor cells as with approved therapeutic radiopharmaceuticals.

Commonly, FAP-targeting radioconjugates suffer from low tumor retention that may consequently constrain their therapeutic efficacy [10]. Therefore, new FAPIs were developed with the intention to enhance tumor uptake and retention. These included OncoFAP, as well as the cyclic peptides FAP-2286 and 3BP-3940 [11–13], which have been evaluated in multiple preclinical studies [10]. Some of the ^177^Lu-labeled analogues were also investigated in clinical settings [14, 15], and in on-going clinical trials to assess safety and efficacy. These include the Phase I trials with [^177^Lu]Lu-FAPI-46 (NCT04849247; 04/2021, China), [^177^Lu]Lu-FAP-2286 (LuMIERE; NCT04939610; 06/2021, Switzerland) and the trimeric OncoFAP derivative [^177^Lu]Lu-OncoFAP-23 (TheraTri; NCT06640413; 10/2025, Italy).

At the Paul Scherrer Institute (PSI), we have established the production [16] and preclinical testing of terbium-161 as a potential alternative to the clinically established lutetium-177 [17]. Terbium-161 belongs to the group of radiolanthanides and emits β¯-particles with similar energies to that of lutetium-177 (Eβ¯_average_= 154 and 134 keV, respectively). The co-emission of γ-radiation by both terbium-161 and lutetium-177 further enables SPECT imaging (Eγ = 49 keV, 75 keV and 113 keV, 208 keV, respectively) [18]. In contrast to lutetium-177, terbium-161 also emits a substantial number of short-ranged electrons (conversion and Auger electrons) that are believed to contribute considerably to the absorbed dose of micrometastases and elicit enhanced radiobiological effects [19, 20]. The interest in this radionuclide led to initial clinical application mainly in combination with ligands targeting the prostate-specific membrane antigen, which showed promising outcome in prostate cancer patients [21, 22]. The increasing awareness of terbium-161 and its growing availability, together with the emergence of FAP as a potential target for radionuclide therapy of sarcoma, has led to initial preclinical and clinical studies using FAP-targeting ^161^Tb-based radioconjugates [23, 24] In this context, the question arises whether FAPIs currently used in combination with lutetium-177 would be suitable candidates to be used with terbium-161.

**Fig. 1:**
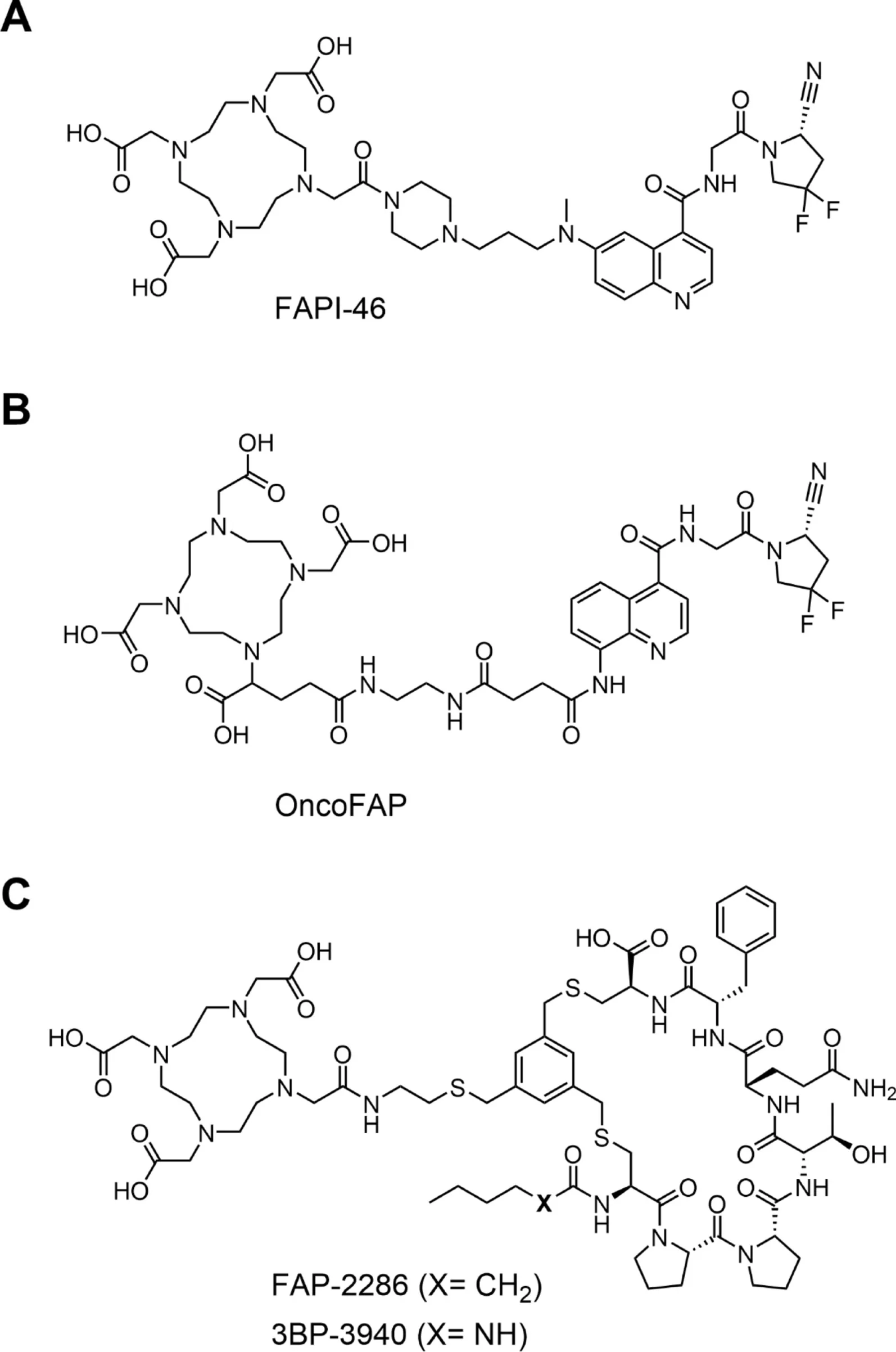
**A–C** Chemical structures of the FAPIs. **A** FAPI-46; **B** OncoFAP; **C** FAP-2286 and 3BP-3940

In this study, four selected FAP-targeting conjugates, FAPI-46 [25], OncoFAP [11], FAP-2286 [12, 24] and 3BP-3940 [13, 26] were labeled with terbium-161 and preclinically evaluated in HT1080-FAP and HT1080 fibrosarcoma cells in vitro and in xenograft-bearing mice for comparison with the data of the ^177^Lu-labeled analogues (Fig. 1). The primary objective was to assess whether FAP-targeting conjugates demonstrate similar biophysical properties and pharmacokinetic behavior when radiolabeled with lutetium-177 and terbium-161. The secondary objective was to evaluate the radioconjugates in terms of FAP-binding affinity, tumor uptake and retention, as well as their tumor-to-background uptake ratios in view of the design of future studies to evaluate their therapeutic efficacy when using terbium-161 in comparison to the use of lutetium-177.

## Materials and methods

### Radiolabeling of FAP-targeting radioconjugates and stability in formulation

The FAPIs FAPI-46, FAP-2286 and 3BP-3940 were purchased from MedChemExpress (USA). OncoFAP was synthesized in-house according to the procedure published by Millul et al. (Supplementary Information; Table S1, Scheme S1, Fig. S1) [11]. No-carrier-added terbium-161 ([^161^Tb]TbCl_3_ in HCl 0.05 M) was obtained from the Radionuclide Development group at PSI [16] or from the company TerThera, Terbium Theranostics (The Netherlands), via Medisynt AG (Switzerland). No-carrier-added lutetium-177 ([^177^Lu]LuCl_3_ in HCl 0.04 M) was obtained from ITM Medical Isotopes GmbH (Germany). Radiolabeling of the FAPIs was performed in a mixture of 0.05 M HCl and 0.5 M sodium acetate at pH ~ 4.5. l-Ascorbic acid was added to the reaction mixtures of all FAP-targeting agents and, in the cases of FAP-2286 and 3BP-3940, also the addition of l-methionine was necessary. The respective radiolabeling mixture was heated at 75–90 °C for 15 min, depending on the FAPI. Quality control was performed using high-performance liquid chromatography (Supplementary Information; Fig. S2).

The stability of the radioconjugates (50 MBq/nmol) in saline (0.9% NaCl) at an activity concentration of 500 MBq/mL was investigated after 1 h, 4 h and 24 h incubation at room temperature (Supplementary Information).

### *n*-Octanol/PBS distribution coefficients

Distribution coefficients (logD values) of the FAP-targeting radioconjugates at an activity concentration of 10 MBq/500 µL were determined by a shake-flask method using *n*-octanol and phosphate-buffered saline (PBS) pH 7.4 followed by phase separation. The distribution coefficients were calculated as the logarithm of the ratio of counts per minute (cpm) in the *n*-octanol phase to those in the PBS phase, as previously reported (Supplementary Information) [27].

### Cell culture

The human fibrosarcoma cell line transfected with FAP (HT1080-FAP cells) and its corresponding parental cell line (HT1080 cells) were kindly provided by Prof. Christoph Renner (Hirslanden Clinic, Zurich, Switzerland) [28]. The cells were cultured in RPMI supplemented with glutamine, antibiotics and 10% fetal calf serum. Geneticin (G418, 0.2 mg/mL, BioConcept, Allschwil, Switzerland) was added to the cell culture medium of HT1080-FAP cells to maintain the FAP expression.

### Cellular uptake of the FAP-targeting radioconjugates

Cellular uptake of the FAP-targeting radioconjugates were determined in HT1080-FAP cells and HT1080 cells (3 × 10^5^ cells/well) seeded in poly-D-lysine-coated 12-well plates. After incubation overnight, the respective radioconjugate (37.5 kBq, 0.75 pmol, 25 µL) was added to each well with or without excess UAMC-1110 (100 µM, MedChemExpress, NJ, USA) to block the FAP. The cell samples were incubated at 37 °C and 5% CO_2_ for 2–4 h, or at 4 °C for 2 h. The cells were then rinsed with either PBS to determine the total uptake of the FAP-targeting radioconjugates or with PBS and an acidic stripping buffer for 30 min. Cell samples were lysed with an aqueous solution of NaOH (1 M) and counted for activity with a γ-counter (Wallac Wizard 1480, PerkinElmer, Waltham, MA, USA). The amount of protein in the samples was measured to equalize the measured activity among all wells (Supplementary Information). The results are reported as average ± standard deviation (SD) of 3–4 independent experiments, performed in triplicates.

### FAP-binding affinity

The FAP-binding affinity of the FAP-targeting radioconjugates (5 MBq/nmol) were determined using HT1080-FAP cells (8 × 10^4^ cells/well) seeded in poly-D-lysine-coated 48-well plates. Increasing molar amounts of the FAPIs (0.05–100 nM) were added to the cells, in the absence or presence of UAMC-1110 (50 µM) to block FAP followed by incubation at 4 °C on ice for 30 min, a commonly used experimental condition in determining saturation binding [29]. The measured activity of the specific binding was plotted against the concentration of the added (radio)conjugate and analyzed through non-linear regression using GraphPad Prism (version 10.6.1) (Supplementary Information). The results are reported as average ± SD of 3–4 independent experiments.

### Radioconjugate binding to mouse and human blood plasma proteins

The FAP-targeting radioconjugates (0.3 MBq, 6 pmol, 15 µL) were incubated with mouse blood plasma (150 µL; Lot: 32321, Rockland Immunochemicals Inc., USA) or human blood plasma (150 µL; Stiftung Blutspende SRK Aargau-Solothurn, Switzerland) at 37 °C for 30 min. The free (plasma protein-unbound) radioconjugate fraction was separated from the plasma protein-bound fraction using Amicon filter devices (Amicon Ultra, 0.5 mL, cut-off 10 kDa, Merck Millipore, Carrigtwohill, Ireland). The activity of the plasma protein-bound fraction, as well as in the filtrate and the filter (considered as the non-bound fraction) were measured separately in a γ-counter to determine the percentage of the FAP-targeting radioconjugate bound to mouse or human plasma proteins (Supplementary Information). The results are reported as average ± SD of 3 independent experiments.

### Blood plasma stability of the FAP-targeting radioconjugates

The stability of the radioconjugates (10 MBq/200 µL) in mouse blood plasma (Lot: 32321, Rockland Immunochemicals Inc., USA) or human blood plasma (Stiftung Blutspende SRK Aargau-Solothurn, Switzerland) was investigated following incubation at 37 °C for up to 24 h, as previously reported (Supplementary Information) [30, 31]. After 1 h, 4 h and 24 h of incubation, aliquots of the respective samples were taken (1 µL, ~ 50 kBq) and placed on reversed-phase thin layer chromatography (TLC) plates. Once the eluent reached the front line, the TLC plates were dried, exposed to a phosphor screen (Super resolution screen PPN 7001486, PerkinElmer, Waltham MA, USA) and visualized using a storage phosphor system (Cyclone Plus, PerkinElmer, Waltham MA, USA). Analysis was performed using the OptiQuanti software (version 5.0, Bright Instrument Co Ltd., PerkinElmer, Waltham MA, USA). The results are reported as average ± SD of 2 independent experiments for each radioconjugate.

### In vivo studies

All international, national and/or institutional guidelines for the care and use of laboratory animals were followed. The studies were performed according to the guidelines of the Swiss regulations for animal welfare after ethics approval by the cantonal committee of animal experimentation and permission by the responsible cantonal authorities (License N^o^ 75721 and related extensions). Five-to-six week-old CD1 nude (Crl: CD1-Foxn^nu^) female mice were obtained from Charles River Laboratories (Sulzfeld, Germany) and acclimatized in the animal house at PSI for at least seven days prior to conducting the studies.

### Dual-isotope SPECT/CT imaging studies

Mice were subcutaneously inoculated with HT1080-FAP cells (5 × 10^6^ cells in 100 µL PBS) and HT1080 cells (3 × 10^6^ cells in 100 µL PBS) on the right and left shoulder, respectively. At least 7 days after inoculation, mice were injected with a mixture of ^161^Tb-based FAPI (10 MBq, 0.25 nmol) and the respective ^177^Lu-labeled counterpart (10 MBq, 0.25 nmol) (*n* = 2 mice for each radioconjugate mixture). Dual-isotope single-photon emission computed tomography/computed tomography (SPECT/CT) scans were performed 1 h, 4 h and 24 h post-injection (p.i.) using a dedicated small-animal SPECT/CT scanner (NanoSPECT/CT, Mediso Medical Imaging Systems, Budapest, Hungary) and the Nucline software (version 1.02, Mediso Ltd., Budapest, Hungary), which was set-up to record two γ-lines of each radionuclide, as previously reported (Supplementary Information) [32].

### Biodistribution studies

Biodistribution studies were performed 7‒10 days after tumor cell inoculation, when the xenografts reached a volume of ~ 100 mm^3^. Mice were injected intravenously with ^161^Tb- or ^177^Lu-labeled FAPI (5 MBq, 0.5 nmol/mouse) in 100 µL saline (0.9% NaCl) containing 0.05% bovine serum albumin (BSA) to prevent unwanted adherence of the radioconjugates to the vial and syringe plastic material. After 1 h, 4 h, or 24 h, the mice were sacrificed and tissues and organs of interest were collected, weighed and counted for activity with a γ-counter. The results were reported as the percentage of the injected activity per tissue mass (% IA/g) using standards of the injection solution measured at the same time to obtain decay-corrected data. The tissue and organ uptake values were calculated as the average ± SD of *n* = 3–5 mice per timepoint. (Supplementary Information).

### Statistical analysis

The results of the in vitro evaluation and biodistribution studies were compared between ^161^Tb-based FAPIs and their ^177^Lu-labeled analogues. The data were analyzed using unpaired t-test with Welch’s correction, which accounted for different variances or sample sizes (GraphPad Prism, version 10.5.0). A *p*-value < 0.05 was considered statistically significant.

## Results

### Radiolabeling and radiolytic stability

Radiolabeling of FAPI-46, OncoFAP, FAP-2286 and 3BP-3940 with terbium-161 and lutetium-177 was achieved at molar activities of 5–50 MBq/nmol, with radiochemical purities of > 98% (Supplementary Information; Fig. S2). Even at high activity concentrations of 500 MBq/mL, all FAP-targeting radioconjugates remained stable in saline, with ≥ 96% intact product after a 24-h incubation period and only marginal signs of radiolytic degradation.

### *n*-Octanol/PBS distribution coefficients

The *n*-octanol/PBS distribution coefficients (logD values) of the ^161^Tb- and ^177^Lu-labeled analogous FAPIs were equal in all four cases (*p* > 0.05). The logD values of all FAP-targeting radioconjugates ranged between − 3 and − 5, with the OncoFAP radioconjugates showing the lowest values and the FAPI-46 radioconjugates displaying the highest (Table 1).

**Table 1:** Summary of the in vitro properties of the FAP-targeting radioconjugates

Radioconjugate	Distribution coefficient (logD values)	FAP-binding affinity (K_D_ values)
	*n* = 3–4	*n* = 3–4
		(nM)
[^161^Tb]Tb-FAPI-46	-3.02 ± 0.01	0.55 ± 0.30
[^177^Lu]Lu-FAPI-46	-3.04 ± 0.03	0.58 ± 0.28
[^161^Tb]Tb-OncoFAP	-4.98 ± 0.07	0.48 ± 0.10
[^177^Lu]Lu-OncoFAP	-5.02 ± 0.01	0.59 ± 0.21
[^161^Tb]Tb-FAP-2286	-3.39 ± 0.03	1.1 ± 0.5
[^177^Lu]Lu-FAP-2286	-3.39 ± 0.13	1.8 ± 0.8
[^161^Tb]Tb-3BP-3940	-3.84 ± 0.07	0.79 ± 0.07
[^177^Lu]Lu-3BP-3940	-3.84 ± 0.10	0.74 ± 0.36

K_D_ results were obtained in cell experiments performed at 4 °C. No statistically significant difference was determined for data of the ^161^Tb- and ^177^Lu-labeled FAPI counterpart (*p* > 0.05)

### Uptake of FAP-targeting radioconjugates in HT1080-FAP and HT1080 cells

**Fig. 2:**
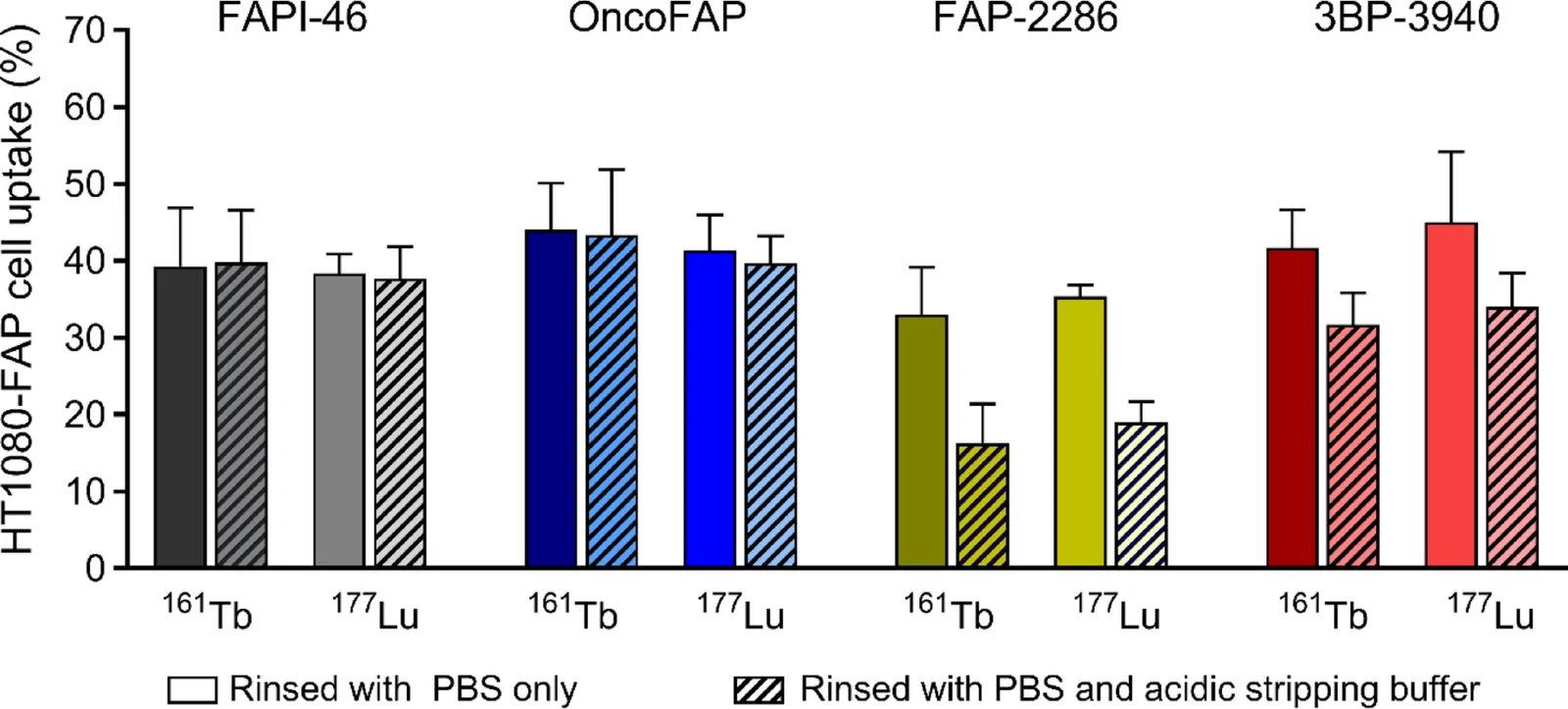
Uptake of the ^161^Tb- and ^177^Lu-labeled FAPIs in HT1080-FAP cells after incubation at 37 °C for 2 h, followed by rinsing of the cells with PBS or acidic stripping buffer. The data are presented as average ± SD of *n* = 3‒4 independent experiments. No statistical difference was determined for data of the respective ^161^Tb- and ^177^Lu-labeled FAPI counterparts (*p* > 0.05)

The ^161^Tb-labeled FAPIs showed a similar uptake in HT1080-FAP cells as their ^177^Lu-based analogues irrespective of whether the cells were rinsed with only PBS or acidic stripping buffer (*p* > 0.05, Fig. 2; Supplementary Information; Fig. S3). After 2 h incubation, the uptake in HT1080-FAP cells was 38–45% for FAPI-46, OncoFAP and 3BP-3940 radioconjugates while the uptake of FAP-2286 radioconjugates reached only 33–35% (Fig. 2). Radioconjugates based on FAPI-46 and OncoFAP showed almost equal uptake when rinsed with PBS or stripped with acidic buffer. Lower measurements were, however, observed for the FAP-2286 and 3BP-3940 radioconjugates following acidic stripping, with values of 16–19% and approximately 32–34%, respectively. After 4 h incubation, the pattern remained the same although the uptake of all radioconjugates marginally increased. Negligible uptake of ≤ 0.1% was observed when the FAP-targeting radioconjugates were co-incubated with excess UAMC-1110 that blocked the FAP-binding site. FAP-targeting radioconjugates were also not taken up by HT1080 cells (< 0.1% after 4 h of incubation) (Supplementary Information; Fig. S4). After 2 h of incubation at 4 °C, the total uptake of the FAP-targeting radioconjugates in HT1080-FAP cells was 20–24% of the added activity, while the measurements were only 12–21% after rinsing the cells with acidic stripping buffer (Supplementary information; Fig. S5).

### FAP-binding affinity of the radioconjugates

The ^161^Tb-labeled FAPIs were characterized with K_D_ values in the low nanomolar range (0.48–1.1 nM), similar to those of the respective ^177^Lu-based analogues (0.58–1.8 nM; *p* > 0.05). The highest FAP-binding affinity was seen with FAPI-46 and OncoFAP radioconjugates, whose K_D_ values ranged from 0.48 nM to 0.59 nM. The highest K_D_ values of 1.1–1.8 nM were obtained for FAP-2286 radioconjugates (Table 1).

### Binding to mouse and human blood plasma proteins

**Fig. 3:**
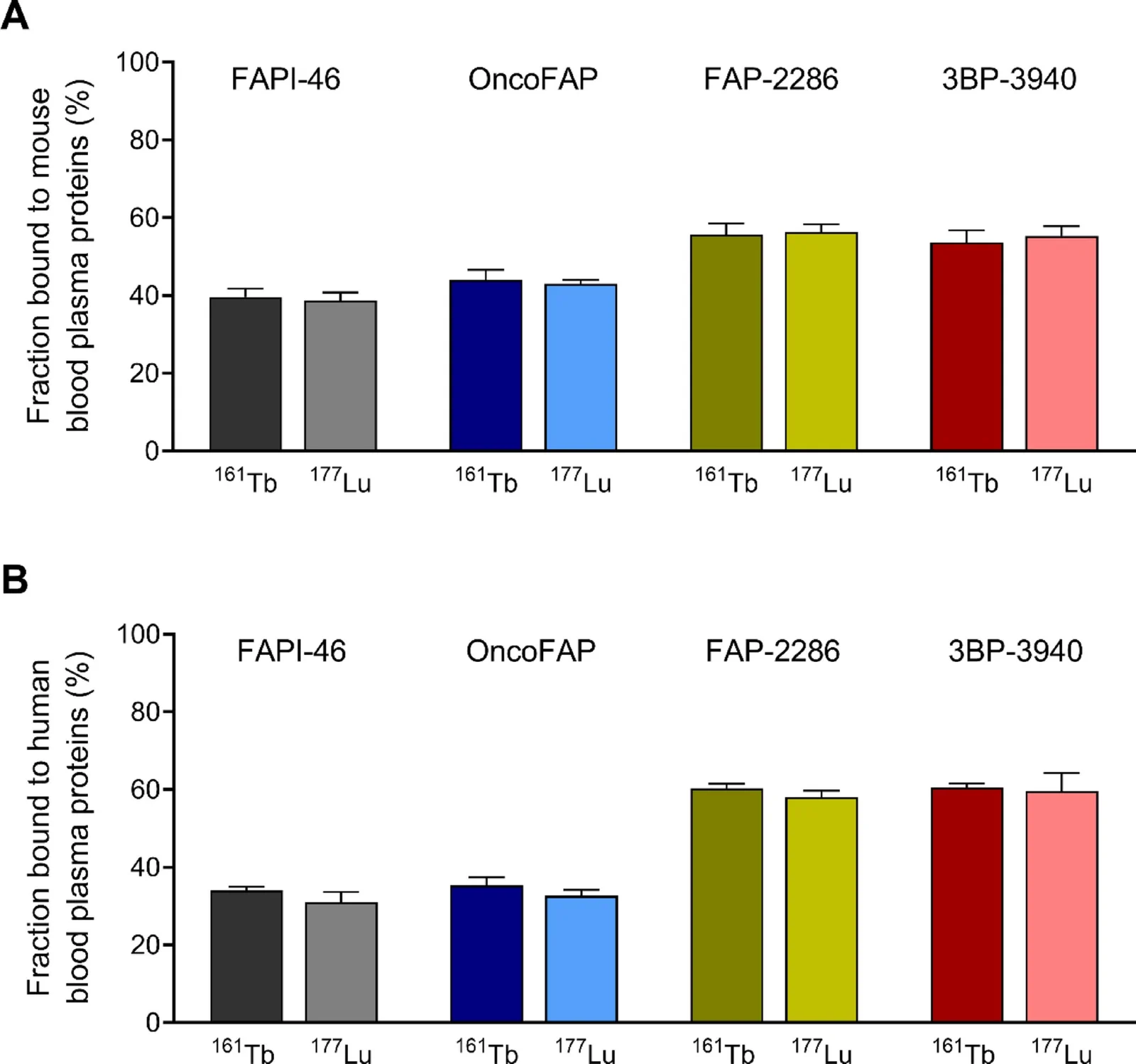
**A/B** Blood plasma protein-bound fractions of the radioconjugates. **A** Plasma protein-bound fractions of ^161^Tb- and ^177^Lu-labeled FAPIs incubated in mouse plasma; **B** Plasma protein-bound fractions of ^161^Tb- and ^177^Lu-labeled FAPIs incubated in human plasma. The data are presented as average ± SD of *n* = 3 independent experiments. No statistical difference was determined for data of respective ^161^Tb- and ^177^Lu-labeled FAPI counterparts (*p* > 0.05)

The blood plasma protein-bound fractions of FAPI-46 and OncoFAP radioconjugates in mouse blood plasma reached 39–44%, whereas that of FAP-2286 and 3BP-3940 radioconjugates were comparable at 54–56% (Fig. 3A; Supplementary Information; Table S2). When the radioconjugates were incubated with human blood plasma, the average blood plasma protein-bound fraction of FAP-2286 and 3BP-3940 radioconjugates reached 58–61%, while the fractions of FAPI-46 and OncoFAP radioconjugates were only 31–34% and 32–36%, respectively (Fig. 3B; Supplementary Information; Table S2). In both mouse and human blood plasma, no significant differences were seen between the plasma protein-bound fractions of the corresponding ^161^Tb- and ^177^Lu-labeled FAPIs (*p* > 0.05).

### Stability in mouse and human blood plasma

In mouse blood plasma, the ^161^Tb- and ^177^Lu-labeled FAPIs remained ≥ 98% stable over the first hour of incubation. After a 24-h incubation period, all FAP-targeting radioconjugates showed still ≥ 87% intact compound (Supplementary Information; Table S3). In human blood plasma, the FAP-targeting radioconjugates were ≥ 97% stable over the first hour of incubation and remained ≥ 91% intact at the 24-h timepoint (Supplementary Information; Table S4).

### Dual-isotope SPECT/CT imaging studies

**Fig. 4:**
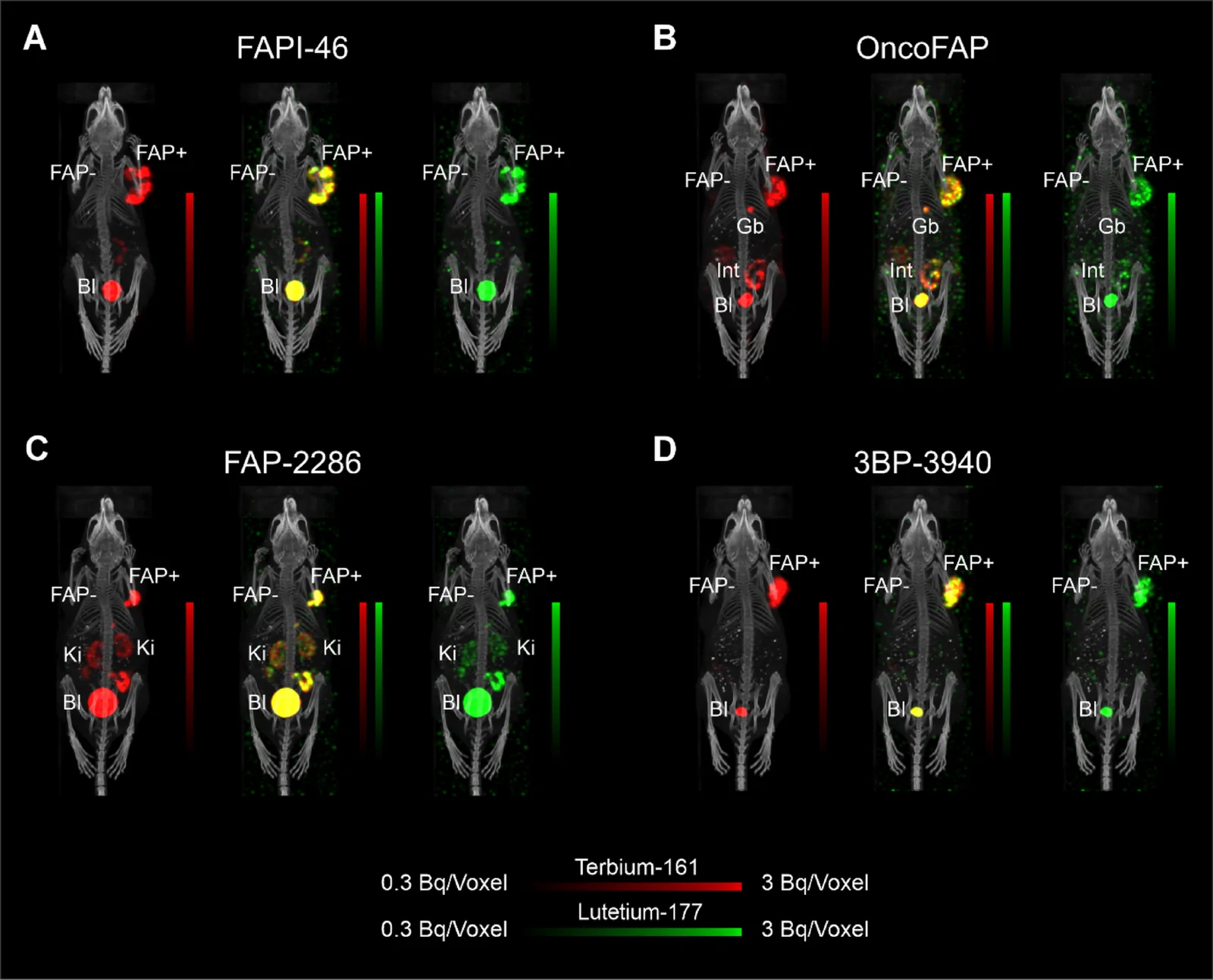
**A–D** Dual-isotope SPECT/CT images of mice bearing HT1080-FAP and HT1080 tumor xenografts at 4 h p.i. of ^161^Tb- and ^177^Lu-labeled FAPIs. **A** [^161^Tb]Tb-FAPI-46 and [^177^Lu]Lu-FAPI-46; **B** [^161^Tb]Tb-OncoFAP and [^177^Lu]Lu-OncoFAP; **C** [^161^Tb]Tb-FAP-2286 and [^177^Lu]Lu-FAP-2286; **D** [^161^Tb]Tb-3BP-3940 and [^177^Lu]Lu-3BP-3940. FAP+: HT1080-FAP tumor; FAP-: HT1080 tumor; Ki: Kidney; Bl: Urinary bladder; Gb: Gall bladder; Int: Intestines. Representative maximum intensity projection images of one mouse from studies performed with *n* = 2 mice per condition

Dual-isotope SPECT/CT images were acquired at 1 h, 4 h and 24 h after injection of mice with a mixture of the ^161^Tb- and ^177^Lu-labeled analogous FAPIs (*n* = 2 mice for each radioconjugate mixture). The images for each radionuclide confirmed the equal tissue distribution of ^161^Tb-based FAPIs and their respective ^177^Lu-labeled equivalents. The radioconjugates accumulated in HT1080-FAP tumors rapidly which enabled the visualization already 1 h after injection, while the uptake in HT1080 tumors was negligible (Supplementary Information; Fig. S6). Renal accumulation was visualized at 1 h after injection of the radioconjugates based on FAPI-46, FAP-2286 and 3BP-3940 but less for OncoFAP. In the cases of FAPI-46 and OncoFAP radioconjugates, some activity was detected in the joints of mice. At the 4-h timepoint, the tumor-to-kidney ratio of accumulated activity was increased in all cases and activity in non-targeted organs and tissue was largely absent (Fig. 4). Traces of activity retention were, however, seen in the intestinal tract and gall blader or in the kidneys after injection of OncoFAP and FAP-2286 radioconjugates, respectively. Images taken 24 h after injection of the FAP-targeting radioconjugates showed that the activity was almost entirely cleared from the bodies of mice, with only marginal HT1080-FAP tumor retention after the injection of 3BP-3940 radioconjugates (Supplementary Information; Fig. S7).

### Biodistribution studies

**Fig. 5:**
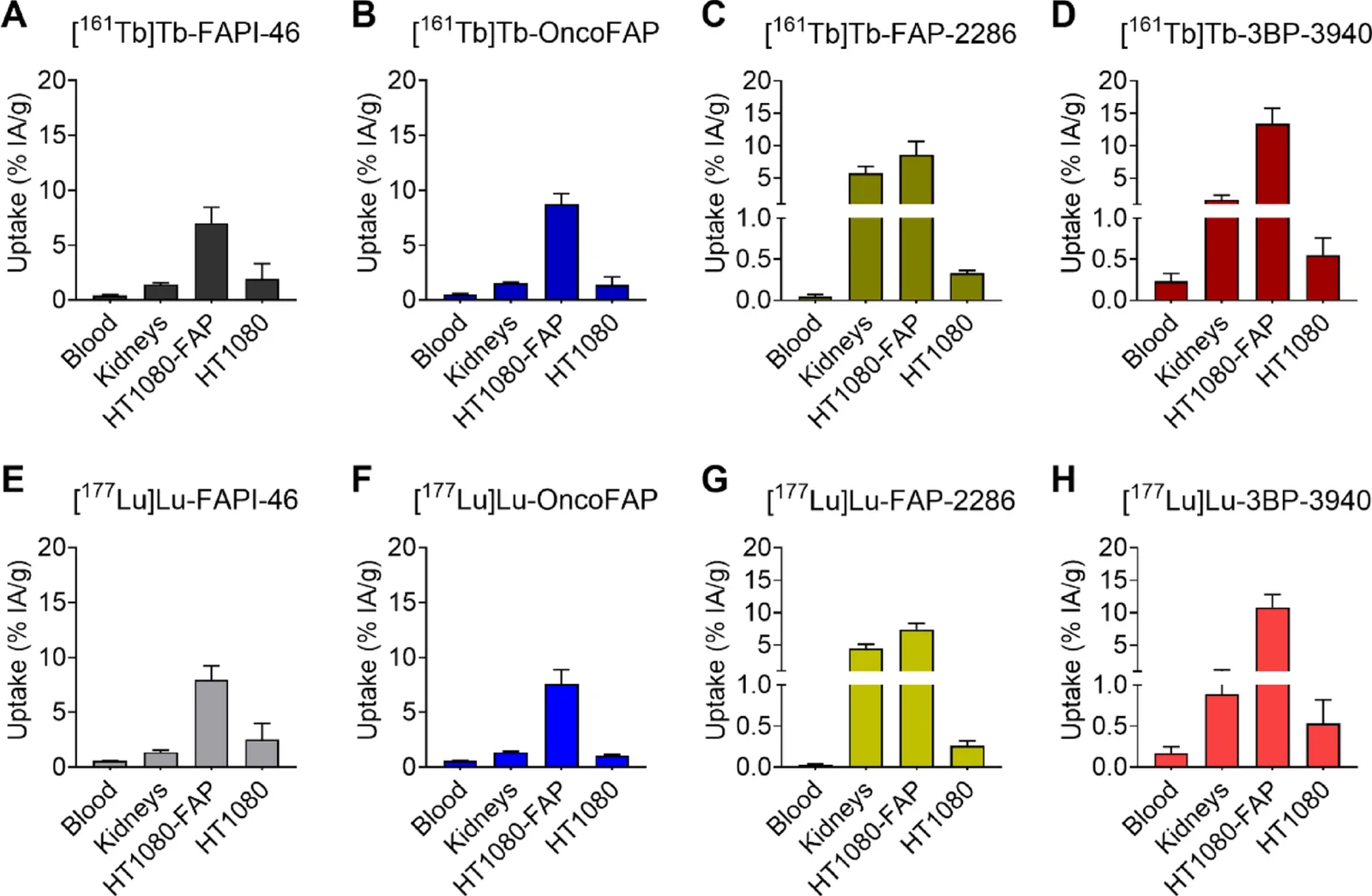
**A–H** Biodistribution data in HT1080-FAP and HT1080 tumor xenografts as well as in blood and kidneys 4 h after injection of the ^161^Tb- and ^177^Lu-labeled FAPIs. **A‒D** ^161^Tb-based FAPIs; **E‒H** ^177^Lu-based FAPIs. **A/E** FAPI-46 radioconjugates; **B/F** OncoFAP radioconjugates; **C/G** FAP-2286 radioconjugates; **D/H** 3BP-3940 radioconjugates. The data are presented as average ± SD of *n* = 3‒5 mice. Analysis of the uptake in HT1080-FAP tumors, HT1080 tumors and kidneys showed no statistical difference between the data obtained for ^161^Tb- and ^177^Lu-labeled FAPI counterparts (*p* > 0.05)

The uptake in HT1080-FAP and HT1080 tumors at 4 h after injection of the ^161^Tb-labeled FAPIs were similar to those of their respective ^177^Lu-based analogues (*p* > 0.05; Fig. 5) (*n* = 3–5 mice per timepoint per radioconjugate). The uptake in other tissues and organs was also comparable between ^161^Tb- and ^177^Lu-based analogues, and no significant differences were determined in the corresponding HT1080-FAP tumor-to-organ uptake ratios (Supplementary Information; Tables S5–S8).

**Fig. 6:**
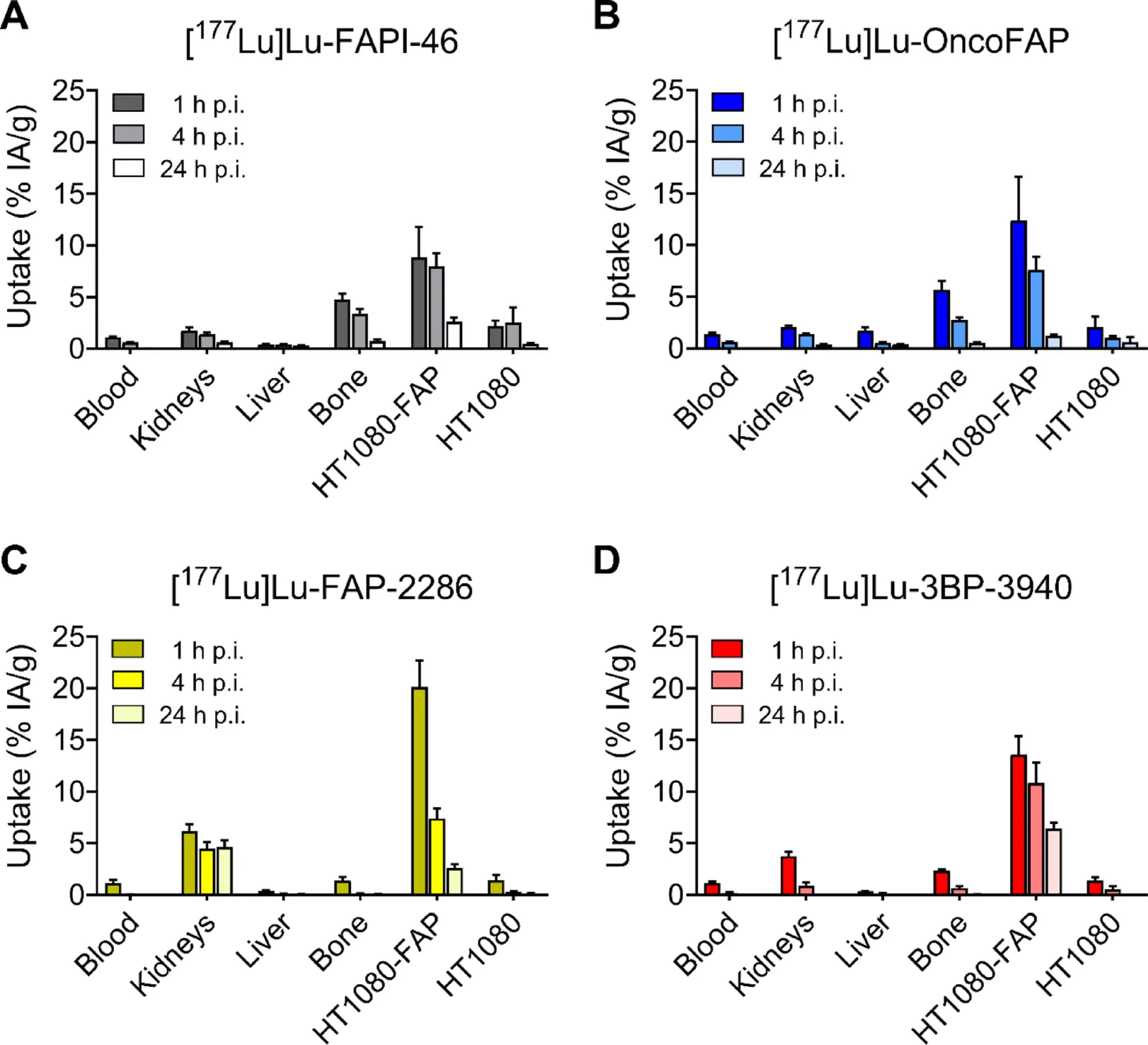
**A–D** Time-dependent biodistribution of ^177^Lu-labeled FAP inhibitors. **A** [^177^Lu]Lu-FAPI-46; **B** [^177^Lu]Lu-OncoFAP; **C** [^177^Lu]Lu-FAP-2286; **D** [^177^Lu]Lu-3BP-3940. The data are presented as average ± SD of *n* = 3‒5 mice

Time-dependent biodistribution data revealed differences among the four investigated FAP-targeting radioconjugates (Supplementary Information; Tables S9–S11). The activity in the blood was similar for all four FAP-targeting radioconjugates with only 1.1–1.4% IA/g at 1 h p.i. and < 0.05% IA/g at the 24-h timepoint (Fig. 6; Supplementary Information; Table S9‒11). The [^177^Lu]Lu-FAPI-46 showed modest uptake of 8.8 ± 2.9% IA/g in the HT1080-FAP tumor xenograft at 1 h p.i. followed by a rapid washout resulting in only 2.6 ± 0.4% IA at 24 h p.i. (Fig. 6A). The time-dependent HT1080-FAP tumor uptake of [^177^Lu]Lu-OncoFAP was similar with slightly higher accumulation of 12 ± 4% IA/g at 1 h p.i. which also declined considerably to only 1.2 ± 0.1% IA/g at 24 h p.i. (Fig. 6B). At the 4-h timepoint, [^177^Lu]Lu-FAPI-46 and [^177^Lu]Lu-OncoFAP had comparable average HT1080-FAP tumor uptake of 7.6–8.0% IA/g.

The HT1080-FAP tumor accumulation of [^177^Lu]Lu-FAP-2286 reached 20 ± 3% IA/g at 1 h p.i., which was followed by a rapid washout to 7.4 ± 1.0% IA/g and 2.6 ± 0.4 24% IA/g measured at 4 h and 24 h p.i., respectively (Fig. 6C). The uptake of [^177^Lu]Lu-3BP-3940 in HT1080-FAP tumors reached 14 ± 2% IA/g at 1 h p.i. however, in this case, the activity was retained better resulting in still 6.4 ± 0.6% IA/g at 24 h p.i.. The uptake of all four FAP-targeting radioconjugates in HT1080 tumors reached only 1.4–2.2% IA/g at 1 h p.i., which declined to background values over the following day (Fig. 6; Supplementary Information; Tables S9–S11).

All four FAP-targeting radioconjugates accumulated in the kidneys at 1 h p.i., resulting in renal uptake of 1.8%–3.7% IA/g, followed by rapid washout to < 1% IA/g at 24 h p.i.. Kidney retention was only observed after the application of [^177^Lu]Lu-FAP-2286, with still 4.6 ± 0.7% IA/g in the kidneys at the 24-h period. Liver uptake was low in all cases and was < 0.5% IA/g at 24 h p.i.. Uptake in the bones, particularly the knee and femur, was considerable at the 1 h p.i. timepoint, followed by decline in activity over time. While [^177^Lu]Lu-FAPI-46 and [^177^Lu]Lu-OncoFAP reached values in the range of 4.7–5.6% IA/g at 1 h p.i., the uptake of [^177^Lu]Lu-FAP-2286 and [^177^Lu]Lu-3BP-3940 reached only 1.4 ± 0.4% IA/g and 2.3 ± 0.1% IA/g, respectively (Fig. 6; Supplementary Information; Table S9).

## Discussion

The findings of this study clearly demonstrated comparable in vitro biophysical properties irrespective of whether the investigated FAPIs were labeled with lutetium-177 or terbium-161. In vivo, ^177^Lu/^161^Tb-based dual-isotope SPECT/CT imaging studies visualized the equal tissue distribution profiles of the corresponding ^161^Tb- and ^177^Lu-labeled FAPIs, which was also confirmed in quantitative biodistribution studies. Equal biophysical and pharmacokinetic properties among corresponding ^161^Tb- and ^177^Lu-based compounds was expected based on previous observation with radioligands targeting the folate receptor [33], somatostatin receptor [32] and prostate-specific membrane antigen [34], among others [35]. Our findings suggest that FAPIs used clinically with lutetium‑177 can also be combined with terbium‑161, without altering tissue distribution properties but with anticipated enhanced therapeutic efficacy.

FAPI-46 belongs to the earlier generation of FAPIs [25]. The modification of the FAP-targeting entity, as seen in the case of OncoFAP was hypothesized to enhance tumor cellular uptake and retention [11]. The conformational rigidity of the cyclic peptides FAP-2286 and 3BP-3940 were also thought to improve the in vivo stability [12, 13]. It remains unclear, however, whether the UAMC-1110 based FAPI-46 and OncoFAP were efficiently internalized by the cells, as reported by Millul et al. [36]. Much more it appears that the acidic stripping buffer does not remove FAP-bound radioconjugates from the surface. The situation with the cyclic peptides slightly differed, as a part of the surface-bound radioconjugates were effectively stripped with acidic buffer. All FAP-targeting radioconjugates, irrespective of their structural differences, showed high FAP-binding affinity with K_D_ values in the low nanomolar range, which was in agreement with prior reports [12]. Radioconjugates based on FAP-2286 and 3BP-3940 showed a 1.3–1.9-fold increased binding to mouse and human blood plasma proteins compared to FAPI-46 and OncoFAP radioconjugates. The in vitro plasma protein binding of these FAP-targeting radioconjugates was, nevertheless, comparable to that of radiopharmaceuticals without designated plasma protein binders [34]. Indeed, in vivo studies showed that the FAP-targeting radioconjugates were rapidly cleared out from the blood.

The rapid wash-out of activity from the tumor, which limited the therapeutic efficacy in preclinical studies performed by other groups [24, 37], was confirmed by our studies. Among the four herein investigated FAPIs, the 3BP-3940 radioconjugates showed a trend of better tumor accumulation and tumor-to-organ uptake ratios. However, even in this case, most activity was cleared from the tumor tissue over 24 h after radioconjugate application, as previously reported [26]. Repeated application and/or higher quantities of activity may be necessary to reach sufficient dose and, hence, therapeutic efficacy in mice and the same may hold true for patients. This is presumably also the reason why it is foreseen to apply ~ 9.25 GBq [^177^Lu]Lu-FAP-2286 per cycle in the planned LuMIERE Phase II trial [38].

Current efforts to enhance tumor uptake and retention of FAPIs and improve the overall tissue distribution are certainly worthwhile with respect to future clinical applications. These strategies include chemical modifications using plasma protein-binding entities [39], the development of multimeric FAPIs [40, 41] and the design of conjugates capable of covalently binding to FAP [42].

Renal uptake was more pronounced with FAP-2286 radioconjugates than with the other three FAPIs, as also observed in other studies [12, 24] and similar to other peptide-based radiopharmaceuticals [43, 44]. Accumulation in the bones, particularly in the joints, was more prominent with the UAMC-1110-based radioconjugates than with the peptide-based FAPIs, as previously reported [36]. The mechanisms underlying this effect remain unclear but may be related to differences in FAP-binding sites between UAMC-1110 [45] and cyclic peptide-based radioconjugates, as suggested by Millul et al. [36]. FAP-targeting radioconjugates may also vary in species cross-reactivity, particularly in binding to mouse FAP versus human FAP that is present in the tumor xenograft [46, 47].

Clinical studies commonly showed favorable tolerability of ^177^Lu-based FAPIs [13, 14]. On-going studies in patients with advanced solid tumors, including the Phase 1/2 clinical trials with [^177^Lu]Lu-FAP-2286 and the OncoFAP derivative [^177^Lu]Lu-OncoFAP-23, will provide further insights regarding the efficacy and safety of FAP-targeted radionuclide therapy. In-depth preclinical investigations are expected to illustrate whether the use of ^161^Tb-based FAPIs will result in enhanced therapeutic efficacy over their ^177^Lu-labeled counterparts, as seen in preclinical evaluation of other targeting agents [34, 44]. To date, the only clinical application of a ^161^Tb-labeled FAPI has been performed with [^161^Tb]Tb-FAP-2286 in a patient with non-small cell lung carcinoma [23]. Investigations using ^161^Tb-based FAPIs will show the potential benefit of this novel radionuclide in FAP-targeted radionuclide therapy, which is certainly most relevant for the treatment of patients with FAP-expressing sarcoma.

## Conclusion

This study demonstrated the feasibility of exchanging lutetium-177 with terbium-161 without affecting the biophysical properties and pharmacokinetic profiles of the investigated FAP-targeting radioconjugates. These observations suggest that the currently employed FAPIs labeled with lutetium-177 could also be applied in combination with terbium-161 while expecting pharmacokinetic equivalence. Future preclinical and clinical studies using ^161^Tb-based FAPIs will elucidate the proposed benefit of using terbium-161 for FAP-targeted radionuclide therapy. The observed rapid clearance of FAP-targeted radioconjugates from the HT1080-FAP tumors would, however, require repeated application and high activities of the radioconjugate to reach therapeutic efficacy in mice.

## Supplementary Information

Below is the link to the electronic supplementary material.

Supplementary Material 1

## Data Availability

All preclinical data analyzed in this study are included in this published article and in the Supplementary Information. Additional information or more detailed data are available from the corresponding author on reasonable request.
