# Supplementary material for: Preclinical characterization of FAP-targeting radioconjugates: assessing biophysical and pharmacokinetic equivalence of lutetium-177 and terbium-161
Source: EJNMMI Res. 2026 Jul 29;16:151. doi: 10.1186/s13550-026-01488-8 (PMC13710466; doi:10.1186/s13550-026-01488-8)
Supplement: Supplementary file 1 [file 13550_2026_1488_MOESM1_ESM.docx]

**SUPPLEMENTARY INFORMATION**

**Preclinical characterization of FAP-targeting radioconjugates: assessing biophysical and pharmacokinetic equivalence of lutetium-177 and terbium-161**

Ana Katrina Mapanao^1^, Ecem Selin Adakli^1^, Christian Vaccarin^1^, Fan Sozzi-Guo^1^, Anzhelika N. Moiseeva^1^, Roger Schibli^1,2^, Nicholas P. van der Meulen^1,3^, Cristina Müller^1,2*^, Thera4Care Consortium

1. Center for Radiopharmaceutical Sciences, PSI Center for Life Sciences, Villigen‑PSI, Switzerland

2. Institute of Pharmaceutical Sciences, Department of Chemistry and Applied Biosciences, ETH Zurich, Zurich, Switzerland

3. Laboratory of Radiochemistry, PSI Center for Nuclear Engineering and Sciences, Villigen‑PSI, Switzerland

***Correspondence to**:

Prof. Dr. Cristina Müller

Center for Radiopharmaceutical Sciences ETH-PSI

Paul Scherrer Institute

5232 Villigen-PSI

Switzerland

e-mail: cristina.mueller@psi.ch

phone: +41 56 310 44 54

fax: +41 56 310 28 49

**1. FAP Inhibitors**

FAPI-46, FAP-2286 and 3BP-3940 were purchased from a commercial provider (MedChemExpress, USA). OncoFAP was synthesized at the Center for Radiopharmaceutical Sciences, ETH Zurich, Zurich, Switzerland (Table S1).

**Table S1** FAP inhibitors used in this study

| **Designation** | **Chelator** | **Provider** | **Catalog N°** | **Alternative name** | **Ref.** |
| --- | --- | --- | --- | --- | --- |
| FAPI-46 | DOTA | MedChemExpress (USA) | HY-137331 | DOTA-FAPI-46 | [1] |
| OncoFAP | DOTAGA | In-house synthesized | - | DOTAGA-OncoFAP | [2] |
| FAP-2286 | DOTA | MedChemExpress (USA) | HY-147057 | DOTA-FAP-2286; Rofapitide tetraxetan | [3, 4] |
| 3BP-3940 | DOTA | MedChemExpress (USA) | HY-P10131 | DOTA-3BP-3940 | [5, 6] |

***Synthesis of OncoFAP:***

OncoFAP was synthesized according to the synthetic procedure previously reported by Millul et al. [2]. The synthetic strategy and detailed chemical conditions are reported below (Scheme S1):

**Methods:** ***Synthesis of (S)-8-amino-N-(2-(2-cyano-4,4-difluoropyrrolidin-1-yl)-2-oxoethyl)quinoline-4-carboxamide (3).*** 8-Aminoquinoline-4-carboxylic acid (**1**, 100 mg, 0.53 mmol, 1.0 equiv.) was suspended in dimethylformamide (DMF, 0.9 mL) and dichloromethane (DCM, 4 mL). (*S*)-1-(2-aminoacetyl)-4,4-difluoropyrrolidine-2-carbonitrile hydrochloride (**2**, 132 mg, 0.585 mmol, 1.1 equiv.) and HATU (202 mg, 0.53 mmol, 1.0 equiv.) were added to the suspension, followed by addition of *N,N*-diisopropylethylamine (DIPEA, 371 μL, 2.1 mmol, 4.0 equiv.). The resultant yellow solution was stirred under nitrogen atmosphere at room temperature (RT) for 5 h and the reaction monitored by thin layer chromatography (TLC) using silica gel plates (eluent: DCM/methanol (MeOH) 90:10, *v/v*). Upon completion, the reaction mixture was diluted with DCM (20 mL) and washed successively with Milli-Q water (10 mL) and brine (10 mL). The organic phase was dried over anhydrous MgSO₄, filtered and concentrated under reduced pressure. The crude residue was purified by silica gel chromatography eluting with DCM/MeOH (95:5, *v/v*).

***Synthesis of (S)-4-((4-((2-(2-cyano-4,4-difluoropyrrolidin-1-yl)-2-oxoethyl)carbamoyl)quinolin-8-yl)amino)-4-oxobutanoic acid (4).*** Succinic anhydride (2.6 g, 26 mmol, 50 equiv.) and 4-dimethylaminopyridine (32 mg, 0.26 mmol, 0.5 equiv.) were added to a solution of intermediate **3** (190 mg, 0.53 mmol, 1.0 equiv.) in tetrahydrofuran (10 mL). The reaction mixture was heated at 60 °C under a nitrogen atmosphere for 3 h while the reaction was monitored using TLC (eluent: DCM/MeOH 90:10, *v/v*). After completion, the solvent was removed under reduced pressure and the resulting solid suspended in Milli-Q water (40 mL) and stirred at RT for 30 min. The solid was collected by filtration and purified by silica gel chromatography using DCM/MeOH (90:10, v/v) as the eluent.

***Synthesis of 2,2',2''-(10-(1-carboxy-4-((2-(4-((4-((2-((S)-2-cyano-4,4-difluoropyrrolidin-1-yl)-2-oxoethyl)carbamoyl)quinolin-8-yl)amino)-4-oxobutanamido)ethyl)amino)-4-oxobutyl)-1,4,7,10-tetraazacyclododecane-1,4,7-triyl)triacetic acid (OncoFAP).*** Intermediate **4** (15 mg, 32 μmol, 1.0 equiv.) was dissolved in dry dimethyl sulfoxide (400 μL), followed by the addition of *N*-hydroxysuccinimide (4.5 mg, 39 μmol, 1.2 equiv.) and *N,N′-*dicyclohexylcarbodiimide (8.8 mg, 42 μmol, 1.3 equiv.). The reaction mixture was stirred overnight at RT in the dark. The following day, the solution was filtered through a 0.45 μm polytetrafluoroethylene (PTFE) filter and the filtrate added to a solution of DOTAGA-NH₂ (20 mg, 39 μmol, 1.2 equiv.) in PBS (100 μL, pH 7.4). The mixture was stirred at RT for 5 h followed by dilution with PBS (1 mL) and a further filtration through a 0.45 μm PTFE filter. The subsequent purification was performed using a semipreparative high performance liquid chromatography (HPLC) system (Merck-Hitachi LaChrom system, Darmstadt, Germany; including a D-7000 interface, L-7200 autosampler, L-7400 UV-Vis detector, L-7100 pump) equipped with a reversed-phase C18 column (Gemini, 5 μm, 10×250 mm, Waters, USA). The compound was eluted using a linear gradient of Milli-Q water containing 0.1% TFA (95% – 60%) and acetonitrile (ACN, 5% – 40%) over 20 min with a flow rate of 4 mL/min. The desired product was detected by evaluating the eluate absorbance at λ=254 nm. The product-containing fractions were collected and combined in a Falcon tube, frozen in liquid nitrogen and lyophilized overnight. The chemical identity of the final product (OncoFAP) was confirmed by high-resolution mass spectrometry (HRMS) analysis (Daltonics maXis II ESI-QTOF, Bruker, USA). The chemical purity of the final product (OncoFAP) was determined by analytical HPLC using the same system as described above, equipped with an analytical reversed-phase C18 column (Sunfire^TM^, 5 μm, 4.6×150 mm, Waters, USA).

**Scheme S1** Synthesis scheme to obtain OncoFAP. Reaction conditions: **A**) HATU, DIPEA, DCM, DMF, at RT, 5 h; **B**) Succinic anhydride, DMAP, THF, 60 °C, 3 h; **C**) **1**. DCC, NHS, DMSO, RT, overnight; **2**. DOTAGA-NH₂, PBS, RT, 5 h

The in-house produced OncoFAP as well as the commercially purchased compounds FAPI-46, FAP-2286 and 3BP-3940, were checked and compared for chemical purity using another HPLC system equipped with a reversed-phase C18 column (Xterra^TM^, 5 μm, 4.6×150 mm, Waters, Milford, MA, U.S.A). The compounds were eluted using a linear gradient of Milli-Q water containing 0.1% TFA (95–20%) and ACN (5–80%) over 15 min at a flow rate of 1 mL/min and subsequently detected by determining the eluate absorbance at λ = 254 nm.

**Results:**

***Chemical characterization of intermediate 3.*** Compound **3** was obtained as a yellow solid (190 mg, 0.53 mmol, quantitative yield).

^1^H-NMR (400 MHz, DMSO-*d_6_*): δ[ppm] 8.97 (t, J = 5.8 Hz, 1H), 8.79 (d, J = 4.3 Hz, 1H), 7.47 (d, J = 4.3 Hz, 1H), 7.41 (dd, J = 8.2, 1.4 Hz, 1H), 7.34 (t, J = 7.5, 1H), 6.91 (dd, J = 8.2, 1.4 Hz, 1H), 5.98 (s, 2H), 5.17 (dd, J= 9.4, 2.6 Hz, 1H), 4.36 – 4.24 (m, 1H), 4.20 (t, J = 5.9 Hz, 2H), 4.18 – 4.07 (m, 1H), 2.99 – 2.76 (m, 2H).

^13^C-NMR (101 MHz, DMSO-*d_6_*): δ[ppm] 167.68, 145.35, 145.36, 142.01, 137.60, 128.18, 124.85, 119.04, 117.82, 111.31, 108.84, 53.60, 51.20, 44.22, 44.15, 41.25, 36.40.

^19^F-NMR (376 MHz, DMSO-*d_6_*): δ[ppm] -95.16, -95.81, -103.58, -104.20.

HRMS (ESI): calculated for C_17_H_16_F_2_N_5_O_2_ [M+H]^+^: calculated: 360.1267, found: 360.1264.

***Chemical characterization of intermediate 4.*** Compound **4** was obtained as a yellow solid (147 mg, 60% yield).

^1^H-NMR (400 MHz, DMSO-*d_6_*): δ[ppm] 10.20 (s, 1H), 9.14 (t, J = 6.0 Hz, 1H), 9.02 d, J = 4.3 Hz, 1H), 8.65 (dd, J = 7.8, 1.3 Hz, 1H), 7.99 (dd, J = 8.6, 1.2 Hz, 1H), 7.66 (d, J = 4.5 Hz, 1H), 7.64 (t, J = 8.2 Hz, 1H), 5.18 (dd, J= 9.3, 2.7 Hz, 1H), 4.38 – 4.28 (m, 1H), 4.24 (t, J = 5.8 Hz, 2H), 4.22 – 4.08 (m, 1H), 3.00 – 2.80 (m, 3H), 2.59 (t, J = 6.8 Hz, 2H).

^13^C-NMR (101 MHz, DMSO-*d_6_*): δ[ppm] 174.30, 171.24, 168.26, 167.63, 148.92, 143.08, 138.72, 135.28, 129.84, 128.13, 127.39, 124.68, 118.29, 117.27, 51.66, 44.71, 41.80, 36.86, 32.01, 29.35.

^19^F-NMR (376 MHz, DMSO-*d_6_*): δ[ppm] -95.13, -95.74, -103.65, -104.27.

HRMS (ESI): calculated for C_21_H_20_F_2_N_5_O_5_ [M+H]^+^: calculated: 460.1427, found: 460.1425.

***Chemical characterization of OncoFAP.*** The desired final product was obtained as a light yellow solid (6.2 mg, 20% yield) with a chemical purity of >98%, as determined by UV-Vis HPLC (Fig. S1).

HRMS (ESI): calculated for C_42_H_57_F_2_N_11_O_13_ [M+2H]^2+^: calculated: 480.7047, found: 480.7051.

***Characterization of FAPI-46, FAP-2286 and 3BP-3940.*** The conjugates were purchased and obtained as solid products with chemical purity of >98%, as determined by UV-Vis HPLC (Fig. S1).

**Fig. S1** Representative HPLC chromatograms of the commercially purchased and in-house synthesized FAPIs with the retention time (t_R_) and chemical purity indicated on the chromatogram

**2. Radiolabeling of the FAP inhibitors**

***Purpose:*** The FAP inhibitors (FAPIs) were labeled with terbium-161 and lutetium-177 for preclinical investigations.

***Methods***: No-carrier-added terbium-161 ([^161^Tb]TbCl_3_ in HCl 0.05 M) was obtained from the Radionuclide Development group at PSI [7] or from Terthera, Terbium Theranostics (The Netherlands), via Medisynt AG (Switzerland). No-carrier-added lutetium-177 ([^177^Lu]LuCl_3_ in HCl 0.04 M) was obtained from ITM Medical Isotopes GmbH (Germany). The FAPIs (Table S1)were labeled with terbium-161 or lutetium-177 at molar activities of 5–50 MBq/nmol, following specific radiolabeling conditions that varied among the four FAPIs.

**FAPI-46:** Radiolabeling of FAPI-46 (1 mM stock solution in Milli-Q water) with terbium-161 and lutetium-177 was performed in a 1:1.25 mixture of sodium acetate (0.5 M) and HCl (0.05 M) and the addition of 3 mg l-ascorbic acid. The radiolabeling mixture was incubated at 75 °C for up to 15 min.

**OncoFAP:** Radiolabeling of OncoFAP (1 mM stock solution in Milli-Q water) with terbium-161 and lutetium-177 was performed in a 1:1.25 mixture of sodium acetate (0.5 M) and HCl (0.05 M) and the addition of 3 mg l-ascorbic acid. The radiolabeling mixture was incubated at 90 °C for 15 min.

**FAP-2286 and 3BP-3940:** Radiolabeling of FAP-2286 and 3BP-3940 (1 mM stock solution in Milli-Q water) with terbium-161 and lutetium-177 was performed in a 1:1.25 mixture of sodium acetate (0.5 M) and HCl (0.05 M) with the addition of 3 mg l-ascorbic acid and 2 mg l-methionine. The radiolabeling mixture was incubated at 90 °C for 15 min.

Quality control of the radiolabeled FAPIs was performed after dilution of the radiolabeling solution with Milli-Q water containing penta-sodium diethylenetriamine pentaacetic acid (Na_5_-DTPA; 50 µM) using reversed-phase high performance liquid chromatography (HPLC). The HPLC system (Merck Hitachi LaChrom HPLC) was equipped with a D-7000 interface, a L-7200 autosampler, a radioactivity detector (LB 506 B; Berthold) and a L-7100 pump connected to a C18 column (Xterra^TM^, 5 μm, 4.6×150 mm, Waters, Milford, MA, U.S.A). A linear gradient of ACN (5–80%) and Milli-Q water with 0.1% TFA (95–20%) was used as a mobile phase at a flow rate of 1 mL/min over 15 min.

***Results:*** The FAPIs labeled with terbium-161 or with lutetium-177 were obtained with >98% radiochemical purity and used for in vitro and in vivo studies without further purification (Fig. S2).

**Fig. S2 A–H** Representative HPLC chromatograms with the respective retention time (t_R_) and radiochemical (RC) purity of the FAP-targeting radioconjugates. **A/B** [^161^Tb]Tb-FAPI-46 and [^177^Lu]Lu-FAPI-46; **C/D** [^161^Tb]Tb-OncoFAP and [^177^Lu]Lu-OncoFAP; **E/F** [^161^Tb]Tb-FAP-2286 and [^177^Lu]Lu-FAP-2286; **G/H** [^161^Tb]Tb-3BP-3940 and [^177^Lu]Lu-3BP-3940. Traces of unreacted terbium-161 and lutetium-177 would have been eluted with t_R_ = 3.2 min

**3. Radiolytic stability of FAP-targeting radioconjugates in formulation**

***Purpose:*** The integrity of the FAP-targeting radioconjugates was investigated over time to identify their radiolytic stability in formulation.

***Methods:*** The FAP-targeting radioconjugates (50 MBq/nmol) were diluted in saline (0.9% NaCl) at an activity concentration of 500 MBq/mL. An aliquot of the solution was collected after 1 h, 4 h and 24 h incubation at room temperature to check the integrity of the radioconjugates using a reversed-phase HPLC system. Qualitative assessment of the radiolytic stability was performed by integration of the chromatogram peaks representing the radioconjugate, released radiometal complexed with DTPA (t_R_ = 3.2 min) and potential degradation products of unknown structure. The integrated peak area of the radioconjugates at the initial timepoint (t = 0 h) was set as 100%.

***Results:*** The FAP-targeting radioconjugates were stable in saline and remained with ≥96% intact product after a 24-h incubation period.

**4. Determination of the *n-*octanol/PBS distribution coefficient**

***Purpose:*** The *n*-octanol/PBS distribution coefficients (logD values) were determined as a measure for the hydrophilic or lipophilic character of the radioconjugates.

***Methods:*** The radioconjugates (50 MBq/nmol) were diluted in phosphate-buffered saline (PBS) pH 7.4 to obtain an activity concentration of 10 MBq/500 µL. A sample of this radioconjugate solution (~0.5 MBq, 25 µL, 0.01 nmol) was added to a mixture of PBS pH 7.4 (1475 µL) and *n*-octanol (1500 µL). The vials were vortexed vigorously for 1 min followed by centrifugation (560 rcf, 25 °C, 6 min) for phase separation. Aliquots were taken from each phase and measured in a γ-counter (Wallac Wizard 1480, PerkinElmer, Waltham, MA, USA). Due to the high hydrophilicity of the OncoFAP radioconjugates that led to counts per minute (cpm) measurements in the *n*-octanol phase that were below the linear range of the γ-counter, the protocol was slightly modified and a larger volume of 250 µL radioconjugate solution was added to a mixture of PBS pH 7.4 (1250 µL) and *n*-octanol (1500 µL). The distribution coefficients were calculated as the logarithmic value of the ratio of cpm measured in the *n*-octanol phase relative to the cpm measured in the PBS phase. The results were listed as average ± standard deviation (SD) of the data obtained from 3–4 independent experiments, each performed with five replicates. The data obtained with the ^161^Tb-labeled radioconjugates were compared to that of their corresponding ^177^Lu-labeled counterparts and were analyzed using an unpaired t-test with Welch’s correction (GraphPad Prism, version 10.5.0). A *p*-value <0.05 was considered statistically significant.

***Results:*** The data are reported in the main article.

**5. Cellular uptake of the radiolabeled FAP inhibitors**

***Purpose:*** The studies were performed to determine and compare the uptake of FAP-targeted radioconjugates in fibrosarcoma cells.

***Methods:*** HT1080-FAP cells and HT1080 cells (3 ×10^5^ cells in 2 mL per well) were seeded in poly-D-lysine-coated 12-well plates. After incubation overnight, the radioconjugates (37.5 kBq, 0.75 pmol, 25 μL) were added to each well with or without UAMC-1110 (MedChemExpress, HY-100684, 100 μM) to block the FAP. The cell samples were incubated at 37 °C and 5% CO_2_ for 2 h or 4 h. Additional uptake studies were performed with the cell samples incubated at 4 °C for 2 h. The cells were then rinsed with either PBS pH 7.4 to determine the total uptake of the FAP-targeted radioconjugate, or with PBS and an acidic stripping buffer for 30 min. Cell samples were lysed with an aqueous solution of NaOH (1 M, 1 mL) and counted for activity in a γ-counter (Wallac Wizard 1480, PerkinElmer, Waltham, MA, USA). The amount of protein of the samples was measured to equalize the measured activity among all wells. The data were expressed as the percentage of the radioconjugate uptake after rinsing the cells with PBS only or with PBS and acidic stripping buffer. The results are reported as average ± SD of 3–4 independent experiments, performed in triplicates. The data obtained with the ^161^Tb-labeled radioconjugates were compared to that of their corresponding ^177^Lu-labeled counterparts and were analyzed using an unpaired t-test with Welch’s correction (GraphPad Prism, version 10.5.0). A *p*-value <0.05 was considered statistically significant.

***Results:*** The uptake of the radioconjugates in HT1080-FAP cells after 2 h incubation and rinsing with PBS or acidic stripping buffer, respectively, are reported in the main article. Data obtained after a 4-h incubation period are visualized below (Fig. S3). The uptake of the FAP-targeting radioconjugates in HT1080 cells was negligible (Fig. S4). When incubated at 4 °C for 2 h, the uptake of the radioconjugates after rising the HT1080-FAP cells with PBS or acidic stripping buffer were 20–24% and 12–21%, respectively (Fig. S5).

**Fig. S3** Uptake of ^161^Tb- and ^177^Lu-labeled FAPIs in HT1080-FAP cells after incubation at 37 °C for 4 h, followed by rinsing of the cells with PBS or acidic stripping buffer (n = 3‒4 independent experiments, each performed in triplicate). The data obtained with the ^161^Tb-based FAP-targeting radioconjugates were not statistically different from those of the ^177^Lu-based counterpart (*p* >0.05)

**Fig. S4** Uptake of the FAP-targeting radioconjugates in HT1080 cells after a 4-h incubation period at 37 °C (n = 2–3 independent experiments, each performed in triplicate)

**Fig. S5** Uptake of the ^177^Lu-based FAPIs in HT1080-FAP cells after 2 h incubation at 4°C, followed by rinsing of the cells with PBS or acidic stripping buffer (n = 3 independent experiments, each performed in triplicate)

**6. FAP-binding affinity (K_D_)**

***Purpose:*** The FAP-binding affinity of the FAP-targeting radioconjugates was determined experimentally for comparison of the ^161^Tb- and the ^177^Lu-labeled analogues and to determine potential differences among the different types of FAPIs.

***Methods:*** HT1080-FAP cells were seeded in poly-D-lysine-coated 48-well plates (8 ×10^4^ cells in 0.5 mL per well) using RPMI culture medium with supplements. The cells were incubated at 37 °C and 5% CO_2_ overnight to allow adhesion. The assay was performed on ice and with ice-cold solutions as it is commonly done for saturation binding experiments[8]. After removal of the medium, the HT1080-FAP cells were rinsed with cold PBS (0.5 mL, pH 7.4) followed by the addition of ice-cold RPMI culture medium without supplements (450 µL/well) in the absence or presence of UAMC-1110 (final concentration 50 µM) as a FAP blocking agent. The cells were incubated for 15 min on ice. The respective radioconjugate (5 MBq/nmol) was diluted in cold PBS pH 7.4 at the FAP-targeting conjugate concentrations of 0.5‒1000 nM. A volume of 50 µL from each dilution was added to each well to obtain conjugate concentrations in the range of 0.05‒100 nM. The cells were incubated at 4 °C on ice for 30 min on an orbital shaker before rinsing twice with ice-cold PBS (0.5 mL, pH 7.4). The cells were lysed using an aqueous NaOH solution (1 M, 600 µL) and the lysates were transferred to radioimmunoassay tubes for counting the activity in a γ-counter (Wallac Wizard 1480, PerkinElmer, Waltham, MA, USA). The K_D_ values were determined by plotting the specific binding (total binding minus unspecific binding) against the molar concentration of the added radioconjugate using GraphPad Prism software (version 10.6.1). The results were then expressed as average ± SD of 3–4 independent experiments. The data obtained with the ^161^Tb-based FAP-targeting radioconjugates were compared to that of their corresponding ^177^Lu-labeled analogues and were analyzed using an unpaired t-test with Welch’s correction (GraphPad Prism, version 10.5.0). A *p*-value <0.05 was considered statistically significant.

***Results:*** The results, along with the K_D_ values indicative of the FAP-binding affinity of the radioconjugates, are described and listed in the main article.

**7. Determination of the plasma protein-binding properties**

***Purpose:*** The radioconjugates were investigated in vitro for their binding to blood plasma proteins in mouse of human blood plasma.

***Methods:*** The binding of the radioconjugates to proteins present in mouse blood plasma (Lot: 32321, Rockland Immunochemicals Inc., USA) and human blood plasma (Stiftung Blutspende SRK Aargau-Solothurn, Switzerland) were determined using an ultrafiltration assay as previously reported [9, 10]. A fixed amount of radioconjugate (50 MBq/nmol, ~300 kBq, 15 μL, 0.006 nmol) was added to 150 μL of mouse or human blood plasma, followed by incubation of the samples at 37 °C for 30 min. After cooling down on ice, the samples were loaded on Amicon centrifugal filters (cut-off of 10 kDa; Merck Millipore, Carrigtwohill, Ireland) and centrifuged (14000 rcf at 4 °C for 30 min) to separate the plasma protein-bound from the free (protein unbound) fractions of each sample. The inserts of the filter device were placed inverted in a clean collection tube and spun at 200 rcf for 3 min to recover the plasma protein-bound fraction. The activities of the protein-unbound radiopeptides in the filtrate, as well as in the filter insert and the plasma protein-bound radiopeptides (A_bound_), were counted for activity separately in a γ-counter (Wallac Wizard 1480, PerkinElmer, Waltham, MA, USA). The total activity (A_total_) was determined as the sum of the activities in the filtrate, filter insert and protein-bound fractions (A_bound_). The percentage of radioconjugate bound to mouse plasma proteins and human plasma proteins was calculated as A_bound_/A_total_*100 and the results were presented as average ± SD of 3 independent experiments. The data obtained with the ^161^Tb-labeled radioconjugates were compared to that of their corresponding ^177^Lu-labeled counterparts and were analyzed using an unpaired t-test with Welch’s correction (GraphPad Prism, version 10.5.0). A *p*-value <0.05 was considered statistically significant.

***Results:*** The data obtained using the FAP-targeting radioconjugates are reported in the main article. The detailed numbers of the amount of protein-bound fraction and relative binding of the radioconjugates to the proteins in mouse and human blood plasma are listed in Table S2.

**Table S2** Protein-bound fraction and relative binding of the ^161^Tb-labeled FAPIs and ^177^Lu-labeled FAPIs to mouse or human plasma proteins

| **Radioconjugate** | **Binding to**  **mouse plasma proteins** | **Binding to**  **human plasma proteins** |
| --- | --- | --- |
|  | n = 3 | n = 3 |
|  | (% Bound fraction) | (% Bound fraction) |
| [^161^Tb]Tb-FAPI-46 | 40 ± 2 | 34 ± 1 |
| [^177^Lu]Lu-FAPI-46 | 39 ± 2 | 31 ± 3 |
| [^161^Tb]Tb-OncoFAP | 44 ± 3 | 35 ± 2 |
| [^177^Lu]Lu-OncoFAP | 43 ± 1 | 33 ± 1 |
| [^161^Tb]Tb-FAP-2286 | 56 ± 3 | 60 ± 1 |
| [^177^Lu]Lu-FAP-2286 | 56 ± 2 | 58 ± 2 |
| [^161^Tb]Tb-3BP-3940 | 54 ± 3 | 60 ± 1 |
| [^177^Lu]Lu-3BP-3940 | 55 ± 3 | 60 ± 5 |

No statistical difference was calculated between the results of ^161^Tb-labeled FAPI and that of its ^177^Lu-based counterpart (*p* >0.05)

**8. In vitro stability in mouse and human plasma**

***Purpose:*** The stability of the radioconjugates was assessed in mouse and human blood plasma as an indication of potential in vivo metabolism of the FAP-targeting radioconjugates.

***Methods:*** A sample of ^161^Tb- or ^177^Lu-based FAPIs was added to mouse blood plasma (Lot: 32321, Rockland Immunochemicals Inc., USA) or human blood plasma (Stiftung Blutspende SRK Aargau-Solothurn, Switzerland) to obtain an activity concentration of 10 MBq/200 µL. The samples were incubated at 37 °C for up to 24 h. Control samples referred to the FAP-targeting radioconjugates diluted in saline at an activity concentration of 10 MBq/200 µL. Aliquots of each sample (1 µL, ~50 kBq) were taken after 1 h, 4 h and 24 h and investigated by means of reversed-phase thin layer chromatography (TLC). TLC Silica gel plates (TLC Silica gel 60 RP-18 F_254_s MERCK Analytical Chromatography) were used as stationary phase and ACN/citrate buffer (4:6; *v*/*v*; pH 5.5) as mobile phase. After the eluent reached the front line, the TLC plates were dried and exposed to a phosphor screen (Super resolution screen PPN 7001486, PerkinElmer, Waltham MA, USA) for 1 min. The respective screen was developed using a storage phosphor system (Cyclone Plus, PerkinElmer, Waltham MA, USA) and analyzed using the OptiQuant Software (version 5.0, Bright Instrument Co Ltd., PerkinElmer, Waltham MA, USA). The data of the radiolytic stability tests and the protein-bound fractions determined in vitro resulted in similar outcomes irrespective of whether the FAPI was labeled with lutetium-177 or terbium-161. It was, thus, assumed that the outcome of the in vitro blood plasma stability studies would also be equal for analogous FAPIs either labeled with terbium-161 or lutetium-177. A total of n = 4 independent experiments (n = 2 for the ^177^Lu-labeled FAPI and n = 2 for ^161^Tb-based analogue) were performed. The data are listed as the average ± SD of n = 2 experiments. The data obtained with the ^161^Tb-labeled radioconjuagtes were compared to that of their corresponding ^177^Lu-labeled counterparts and were analyzed using an unpaired t-test with Welch’s correction (GraphPad Prism, version 10.5.0). A *p*-value <0.05 was considered statistically significant.

***Results:*** The results are described in the main article and listed in Tables S3/S4.

**Table S3** Stability data of the FAP-targeting radioconjugates in mouse blood plasma in vitro

| Radioconjugate |  | Intact radioconjugate in mouse blood plasma (%) | | |
| --- | --- | --- | --- | --- |
|  |  | 1 h | 4 h | 24 h |
| [^161^Tb]Tb-FAPI-46 | n = 2 | ≥98 | ≥98 | 97 ± 1 |
| [^177^Lu]Lu-FAPI-46 | n = 2 | ≥98 | ≥98 | 96 ± 1 |
| [^161^Tb]Tb-OncoFAP | n = 2 | ≥98 | ≥98 | 87 ± 6 |
| [^177^Lu]Lu-OncoFAP | n = 2 | ≥98 | ≥98 | 90 ± 1 |
| [^161^Tb]Tb-FAP-2286 | n = 2 | ≥98 | 94 ± 1 | 89 ± 0 |
| [^177^Lu]Lu-FAP-2286 | n = 2 | ≥98 | 96 ± 2 | 91 ± 1 |
| [^161^Tb]Tb-3BP-3940 | n = 2 | ≥98 | ≥98 | 92 ± 2 |
| [^177^Lu]Lu-3BP-3940 | n = 2 | ≥98 | 97 ± 5 | 95 ± 1 |

No statistical difference was calculated between the results of ^161^Tb-labeled FAPI and that of its ^177^Lu-based counterpart (*p* >0.05)

**Table S4** Stability data of the FAP-targeting radioconjugates in human blood plasma in vitro

| Radioconjugate |  | Intact radioconjugate in human blood plasma (%) | | |
| --- | --- | --- | --- | --- |
|  |  | 1 h | 4 h | 24 h |
| [^161^Tb]Tb-FAPI-46 | n = 2 | ≥98 | ≥98 | ≥98 |
| [^177^Lu]Lu-FAPI-46 | n = 2 | ≥98 | ≥98 | ≥98 |
| [^161^Tb]Tb-OncoFAP | n = 2 | ≥98 | ≥98 | 91 ± 5 |
| [^177^Lu]Lu-OncoFAP | n = 2 | ≥98 | ≥98 | 93 ± 0 |
| [^161^Tb]Tb-FAP-2286 | n = 2 | 97 ± 4 | ≥98 | ≥98 |
| [^177^Lu]Lu-FAP-2286 | n = 2 | ≥98 | ≥98 | ≥98 |
| [^161^Tb]Tb-3BP-3940 | n = 2 | ≥98 | ≥98 | ≥98 |
| [^177^Lu]Lu-3BP-3940 | n = 2 | ≥98 | ≥98 | ≥98 |

No statistical difference was calculated between the results of ^161^Tb-labeled FAPI and that of its ^177^Lu-based counterpart (*p* >0.05)

**9. Dual-isotope SPECT/CT imaging**

***Purpose:*** Single photon emission computed tomography/computed tomography (SPECT/CT) imaging studies were performed to determine the whole-body tissue distribution profile of the radioconjugates and determine as to whether the ^161^Tb- and ^177^Lu-labeled version of each FAPI shows the same pharmacokinetic properties.

***Methods:*** Mice were subcutaneously inoculated with HT1080-FAP cells (5 ×10^6^ cells in 100 µL PBS) on the right shoulder and HT1080 cells (3 ×10^6^ cells in 100 µL PBS) on the left shoulder. Dual-isotope SPECT/CT imaging studies were performed approximately 7 d later, when the xenografts reached a volume of at least 100 mm^3^. The FAPIs were labeled with terbium-161 or lutetium-177 at 40 MBq/nmol. DTPA (5 mM) was added after radiolabeling to complex unreacted radiometals. HT1080-FAP/HT1080 tumor-bearing mice (n = 2 for each radioconjugate mixture) were injected with 20 MBq (0.5 nmol) of a mixture of ^161^Tb-based FAPI (10 MBq, 0.25 nmol) and its ^177^Lu-labeled counterpart (10 MBq, 0.25 nmol) prepared in 0.05% bovine serum albumin (BSA). A total of n=8 mice were employed for the dual-isotope SPECT/CT imaging studies. The SPECT/CT images were acquired 1 h, 4 h and 24 h after injection of the activity solutions using a dedicated small-animal SPECT/CT scanner (NanoSPECT/CT, Mediso Medical Imaging Systems, Budapest, Hungary) and the Nucline software (version 1.02, Mediso Ltd., Budapest, Hungary). CT scans of 7.5 min duration were followed by SPECT scans of ~50 min.

Counts stemming from the γ-lines and x-rays of terbium-161 (47.7 keV ± 10% and 74.6 keV ± 10%) and the γ-lines of lutetium-177 (112.9 keV ± 10% and 208.4 ± 10%) were simultaneously registered using the respective energy windows for each radionuclide. Data were reconstructed based on either the

γ-lines and x-rays of terbium-161 or the γ-lines of lutetium-177 to obtain separate distribution profiles for each radioconjugate as previously reported [11]. Real-time CT reconstruction used a cone-beam-filtered back projection and reconstruction of the SPECT data was performed using the HiSPECT software (version 1.4.3049, Scivis GmbH, Göttingen, Germany). Images were prepared using VivoQuant post-processing software (version 3.5, inviCRO Imaging Services and Software, Boston, MA, USA). A Gaussian post-reconstruction filter (full width at half maximum = 1.0 mm) was applied and the scale of activity was set as indicated on the images (0.3‒3 Bq/Voxel).

***Results:*** Dual-isotope SPECT/CT images obtained at 4 h p.i. of the FAP-targeting radioconjugates are reported and described in the main article. Results visualized at 1 h and 24 h p.i. are shown in Figs. S6/S7.

**Fig. S6 A–D** Dual-isotope SPECT/CT images of mice bearing HT1080-FAP and HT1080 tumor xenografts at 1 h p.i. of ^161^Tb- and ^177^Lu-labeled FAPIs. **A** [^161^Tb]Tb-FAPI-46 and [^177^Lu]Lu-FAPI-46; **B** [^161^Tb]Tb-OncoFAP and [^177^Lu]Lu-OncoFAP; **C** [^161^Tb]Tb-FAP-2286 and [^177^Lu]Lu-FAP-2286; **D** [^161^Tb]Tb-3BP-3940 and [^177^Lu]Lu-3BP-3940. FAP+: HT1080-FAP tumor; FAP-: HT1080 tumor; Ki: Kidney; Bl: Urinary bladder; J: Joints; Gb: Gall bladder. Representative maximum intensity projection images of one of n = 2 mice per condition are shown

**Fig. S7 A–D** Dual-isotope SPECT/CT images of mice bearing HT1080-FAP and HT1080 tumor xenografts at 24 h p.i. of ^161^Tb- and ^177^Lu-labeled FAPIs. **A** [^161^Tb]Tb-FAPI-46 and [^177^Lu]Lu-FAPI-46; **B** [^161^Tb]Tb-OncoFAP and [^177^Lu]Lu-OncoFAP; **C** [^161^Tb]Tb-FAP-2286 and [^177^Lu]Lu-FAP-2286; **D** [^161^Tb]Tb-3BP-3940 and [^177^Lu]Lu-3BP-3940. FAP+: HT1080-FAP tumor; FAP-: HT1080 tumor; Ki: Kidney. Representative maximum intensity projection images of one of n = 2 mice per condition are shown

**10. Biodistribution studies**

***Purpose:*** Biodistribution studies were performed in HT1080-FAP/HT1080 tumor-bearing mice to determine and compare the tissue distribution of the ^161^Tb- and ^177^Lu-labeled counterparts as well as to compare the four FAPIs.

***Methods:*** Mice were subcutaneously inoculated with HT1080-FAP cells (5 ×10^6^ cells in 100 µL PBS) on the right shoulder and HT1080 cells (3 ×10^6^ cells in 100 µL PBS) on the left shoulder. Biodistribution studies were performed approximately 7 d later, when the xenografts reached a volume of at least 100 mm^3^. Mice were intravenously injected with ^177^Lu-labeled FAPI (5 MBq, 0.5 nmol/mouse, 100 µL saline containing 0.05% BSA) and sacrificed at 1 h, 4 h or 24 h post-injection (p.i.). Another group of mice were injected with ^161^Tb-based FAPIs (5 MBq, 0.5 nmol/mouse, 100 µL saline with 0.05% BSA) and sacrificed at 4 h p.i. The tissues and organs of interest were collected, weighed and counted for activity in a γ-counter (Wallac Wizard 1480, PerkinElmer, Waltham, MA, USA). There were initially n = 3 mice for each timepoint. If the variation among the three mice was too high or a mouse only had one of the tumor xenografts (e.g. only HT1080-FAP tumor grew on the right should and not the HT1080 tumor on the left shoulder), additional mice were included for that specific timepoint up to a maximum total of n = 5 mice. A total of 57 mice were employed for the biodistribution studies. The results were reported as the percentage of the injected activity per gram of tissue mass (% IA/g) using standards of the injection solution measured at the same time to obtain decay-corrected data. The injected activity per gram tissue mass (% IA/g) was calculated as the average SD of n = 3–5 mice per timepoint. As the similarities of the tissue distribution of ^161^Tb-based FAPIs and ^177^Lu-based FAPIs were already visualized in the dual-isotope SPECT/CT imaging studies and can also be inferred from a single timepoint, comparison of the quantitative tissue and organ uptake measurements were only performed at a single timepoint of 4 h p.i.. Activity measurements in the kidneys, HT1080-FAP tumor and HT1080 tumor, as well as the HT1080-FAP-to-tissue ratios, after the application of ^161^Tb-based FAPIs were compared to those of their respective ^177^Lu-based counterparts. These data were analyzed using unpaired t-test with Welch’s correction (GraphPad Prism, version 10.5.0). A *p*-value <0.05 was considered statistically significant.

***Results:*** The results are reported and described in the main article. Comparisons of the biodistribution of ^161^Tb-based FAPIs and their respective ^177^Lu-labeled counterparts are shown in Tables S5–S8. The time-dependent biodistribution of ^177^Lu-labeled FAPIs are reported in Tables S9–S11.

**Table S5** Biodistribution data and HT1080-FAP tumor-to-tissue uptake ratios of accumulated activity obtained in HT1080-FAP and HT1080 tumor xenograft-bearing mice at 4 h after injection of [^161^Tb]Tb-FAPI-46 or [^177^Lu]Lu-FAPI-46. Decay-corrected data of accumulated activity are shown as % IA/g tissue, representing the average ± SD of n = 3–4 mice

|  | **Biodistribution of ^161^Tb- and ^177^Lu-labeled FAPI-46** | |
| --- | --- | --- |
|  | [^161^Tb]Tb-FAPI-46 | [^177^Lu]Lu-FAPI-46 |
| **Organ** | n = 4 | n = 3 |
|  | (% IA/g) | (% IA/g) |
| Blood | 0.44 ± 0.06 | 0.59 ± 0.03 |
| Heart | 0.24 ± 0.03 | 0.32 ± 0.05 |
| Lungs | 0.37 ± 0.04 | 0.44 ± 0.01 |
| Spleen | 0.21 ± 0.05 | 0.24 ± 0.01 |
| Kidneys | 1.4 ± 0.2 | 1.4 ± 0.2 |
| Stomach | 0.19 ± 0.03 | 0.31 ± 0.09 |
| Pancreas | 0.46 ± 0.08 | 0.67 ± 0.19 |
| Intestine | 0.23 ± 0.06 | 0.38 ± 0.17 |
| Liver | 0.32 ± 0.03 | 0.40 ± 0.04 |
| Muscle | 0.57 ± 0.11 | 0.74 ± 0.03 |
| Bone | 2.3 ± 0.5 | 3.4 ± 0.5 |
| Brain | <0.05 | <0.05 |
| Salivary glands | 0.79 ± 0.16 | 1.0 ± 0.1 |
| Gall bladder | 0.71 ± 0.12 | 0.77 ± 0.29 |
| HT1080-FAP tumor | 7.0 ± 1.4 | 8.0 ± 1.3 |
| HT1080 tumor | 2.0 ± 1.4^#^ | 2.5 ± 1.5 |
|  |  |  |
|  | [^161^Tb]Tb-FAPI-46 | [^177^Lu]Lu-FAPI-46 |
| **Uptake ratios** | n = 4 | n = 3 |
| HT1080-FAP tumor-to-HT1080 tumor ratio | 4.7 ± 1.9^#^ | 4.0 ± 2.2 |
| HT1080-FAP tumor-to-blood ratio | 16 ± 2 | 14 ± 2 |
| HT1080-FAP tumor-to-kidneys ratio | 4.9 ± 0.7 | 5.8 ± 0.7 |
| HT1080-FAP tumor-to-bone ratio | 3.1 ± 0.6 | 2.4 ± 0.4 |

No significant differences between the results of [^161^Tb]Tb-FAPI-46 and [^177^Lu]Lu-FAPI-46 (*p* >0.05). Analysis was performed in selected organ and tissues (kidneys, HT1080-FAP tumor and HT1080 tumor), as well as HT1080-FAP tumor-to-tissue ratios. ^#^One mouse did not develop HT1080 tumor; hence, n = 3 was considered for the analysis

**Table S6** Biodistribution data and HT1080-FAP tumor-to-tissue uptake ratios of accumulated activity obtained in HT1080-FAP and HT1080 tumor xenograft-bearing mice at 4 h after injection of [^161^Tb]Tb-OncoFAP or [^177^Lu]Lu-OncoFAP. Decay-corrected data of accumulated activity are shown as % IA/g tissue, representing the average ± SD of n = 3–4 mice

|  | **Biodistribution of ^161^Tb- and ^177^Lu-labeled OncoFAP** | |
| --- | --- | --- |
|  | [^161^Tb]Tb-OncoFAP | [^177^Lu]Lu-OncoFAP |
| **Organ** | n = 3 | n = 4 |
|  | (% IA/g) | (% IA/g) |
| Blood | 0.52 ± 0.06 | 0.59 ± 0.05 |
| Heart | 0.29 ± 0.01 | 0.33 ± 0.05 |
| Lungs | 0.45 ± 0.02 | 0.461 ± 0.004 |
| Spleen | 0.26 ± 0.02 | 0.30 ± 0.03 |
| Kidneys | 1.6 ± 0.1 | 1.4 ± 0.1 |
| Stomach | 0.28 ± 0.08 | 0.24 ± 0.01 |
| Pancreas | 0.58 ± 0.02 | 0.64 ± 0.14 |
| Intestine | 0.64 ± 0.24 | 0.60 ± 0.20 |
| Liver | 0.49 ± 0.05 | 0.53 ± 0.05 |
| Muscle | 0.61 ± 0.18 | 0.67 ± 0.18 |
| Bone | 2.5 ± 0.3 | 2.7 ± 0.3 |
| Brain | <0.05 | <0.05 |
| Salivary glands | 1.0 ± 0.1 | 1.1 ± 0.2 |
| Gall bladder | 12 ± 4 | 12 ± 3 |
| HT1080-FAP tumor | 8.7 ± 1.0 | 7.6 ± 1.3 |
| HT1080 tumor | 1.4 ± 0.7 | 1.1 ± 0.1^#^ |
|  |  |  |
|  | [^161^Tb]Tb-OncoFAP | [^177^Lu]Lu-OncoFAP |
| **Uptake ratios** | n = 3 | n = 4 |
| HT1080-FAP tumor-to-HT1080 tumor ratio | 7.8 ± 4.7 | 7.6 ± 2.2^#^ |
| HT1080-FAP tumor-to-blood ratio | 17 ± 3 | 13 ± 2 |
| HT1080-FAP tumor-to-kidneys ratio | 5.7 ± 0.9 | 5.4 ± 0.8 |
| HT1080-FAP tumor-to-bone ratio | 3.6 ± 0.4 | 2.8 ± 0.4 |

No significant differences between the results of [^161^Tb]Tb-OncoFAP and [^177^Lu]Lu-OncoFAP (*p* >0.05). Analysis was performed in selected organ and tissues (kidneys, HT1080-FAP xenograft and HT1080 xenograft), as well as HT1080-FAP xenograft-to-tissue ratios. ^#^One mouse did not develop HT1080 tumor; hence, n = 3 was considered for the analysis

**Table S7** Biodistribution data and HT1080-FAP tumor-to-tissue uptake ratios of accumulated activity obtained in HT1080-FAP and HT1080 tumor xenograft-bearing mice at 4 h after injection of [^161^Tb]Tb-FAP-2286 or [^177^Lu]Lu-FAP-2286. Decay-corrected data of accumulated activity are shown as % IA/g tissue, representing the average ± SD of n = 3 mice

|  | **Biodistribution of ^161^Tb- and ^177^Lu-labeled**  **FAP-2286** | |
| --- | --- | --- |
|  | [^161^Tb]Tb-FAP-2286 | [^177^Lu]Lu-FAP-2286 |
| **Organ** | n = 3 | n = 3 |
|  | (% IA/g) | (% IA/g) |
| Blood | <0.05 | <0.05 |
| Heart | <0.05 | <0.05 |
| Lungs | 0.10 ± 0.03 | 0.08 ± 0.02 |
| Spleen | 0.06 ± 0.02 | <0.05 |
| Kidneys | 5.8 ± 1.1 | 4.5 ± 0.7 |
| Stomach | 0.05 ± 0.01 | <0.05 |
| Pancreas | 0.06 ± 0.02 | <0.05 |
| Intestine | 0.14 ± 0.05 | 0.08 ± 0.04 |
| Liver | 0.12 ± 0.03 | 0.10 ± 0.02 |
| Muscle | 0.05 ± 0.02 | <0.05 |
| Bone | 0.13 ± 0.04 | 0.12 ± 0.01 |
| Brain | <0.05 | <0.05 |
| Salivary glands | 0.09 ± 0.03 | 0.06 ± 0.02 |
| Gall bladder | 3.6 ± 1.6 | 1.1 ± 0.2 |
| HT1080-FAP tumor | 8.6 ± 2.1 | 7.4 ± 1.0 |
| HT1080 tumor | 0.33 ± 0.03 | 0.26 ± 0.06 |
|  |  |  |
|  | [^161^Tb]Tb-FAP-2286 | [^177^Lu]Lu-FAP-2286 |
| **Uptake ratios** | n = 3 | n = 3 |
| HT1080-FAP tumor-to-HT1080 tumor ratio | 26 ± 5 | 30 ± 11 |
| HT1080-FAP tumor-to-blood ratio | 255 ± 162 | 269 ± 96 |
| HT1080-FAP tumor-to-kidneys ratio | 1.5 ± 0.2 | 1.7 ± 0.4 |
| HT1080-FAP tumor-to-bone ratio | 65 ± 9 | 61 ± 12 |

No significant differences between the results of [^161^Tb]Tb-FAP-2286 and [^177^Lu]Lu-FAP-2286 (*p* >0.05). Analysis was performed in selected organ and tissues (kidneys, HT1080-FAP xenograft and HT1080 xenograft), as well as HT1080-FAP xenograft-to-tissue ratios

**Table S8** Biodistribution data and HT1080-FAP tumor-to-tissue uptake ratios of accumulated activity obtained in HT1080-FAP and HT1080 tumor xenograft-bearing mice at 4 h after injection of [^161^Tb]Tb-3BP-3940 or [^177^Lu]Lu-3BP-3940. Decay-corrected data of accumulated activity are shown as % IA/g tissue, representing the average ± SD of n = 5 mice

|  | **Biodistribution of ^161^Tb- and ^177^Lu-labeled**  **3BP-3940** | |
| --- | --- | --- |
|  | [^161^Tb]Tb-3BP-3940 | [^177^Lu]Lu-3BP-3940 |
| **Organ** | n = 5 | n = 5 |
|  | (% IA/g) | (% IA/g) |
| Blood | 0.23 ± 0.10 | 0.17 ± 0.08 |
| Heart | 0.12 ± 0.05 | 0.08 ± 0.04 |
| Lungs | 0.29 ± 0.11 | 0.19 ± 0.06 |
| Spleen | 0.09 ± 0.03 | 0.05 ± 0.02 |
| Kidneys | 1.7 ± 0.7 | 0.89 ± 0.32 |
| Stomach | 0.12 ± 0.06 | 0.07 ± 0.02 |
| Pancreas | 0.15 ± 0.06 | 0.11 ± 0.04 |
| Intestine | 0.19 ± 0.08 | 0.11 ± 0.04 |
| Liver | 0.20 ± 0.06 | 0.13 ± 0.03 |
| Muscle | 0.18 ± 0.09 | 0.17 ± 0.07 |
| Bone | 0.64 ± 0.23 | 0.65 ± 0.17 |
| Brain | <0.05 | <0.05 |
| Salivary glands | 0.27 ± 0.12 | 0.21 ± 0.08 |
| Gall bladder | 2.4 ± 1.1 | 1.7 ± 0.7 |
| HT1080-FAP tumor | 13 ± 2 | 11 ± 2 |
| HT1080 tumor | 0.55 ± 0.21^#^ | 0.53 ± 0.29 |
|  |  |  |
|  | [^161^Tb]Tb-3BP-3940 | [^177^Lu]Lu-3BP-3940 |
| **Uptake ratios** | n = 5 | n = 5 |
| HT1080-FAP tumor-to-HT1080 tumor ratio | 27 ± 9^#^ | 24 ± 9 |
| HT1080-FAP tumor-to-blood ratio | 66 ± 22 | 70 ± 18 |
| HT1080-FAP tumor-to-kidneys ratio | 8.5 ± 2.5 | 13 ± 4 |
| HT1080-FAP tumor-to-bone ratio | 23 ± 7 | 17 ± 2 |

No significant differences between the results of [^161^Tb]Tb-3BP-3940 and [^177^Lu]Lu-3BP-3940 (*p* >0.05). Analysis was performed in selected organ and tissues (kidneys, HT1080-FAP xenograft and HT1080 xenograft), as well as HT1080-FAP xenograft-to-tissue ratios. ^#^One mouse did not develop HT1080 tumor; hence, n = 4 was considered for the analysis

**Table S9** Biodistribution data and HT1080-FAP tumor-to-tissue uptake ratios of accumulated activity obtained in HT1080-FAP and HT1080 tumor xenograft-bearing mice at 1 h after injection of the FAP-targeting radioconjugates labeled with lutetium-177. Decay-corrected data of accumulated activity are shown as % IA/g tissue, representing the average ± SD of n = 3–5 mice

|  | **Biodistribution of ^177^Lu-labeled FAPIs at 1 h p.i.** | | | |
| --- | --- | --- | --- | --- |
|  | [^177^Lu]Lu-  FAPI-46 | [^177^Lu]Lu-OncoFAP | [^177^Lu]Lu-  FAP-2286 | [^177^Lu]Lu-  3BP-3940 |
| **Organ** | n = 5 | n = 4 | n = 3 | n = 3 |
|  | (% IA/g) | (% IA/g) | (% IA/g) | (% IA/g) |
| Blood | 1.1 ± 0.1 | 1.4 ± 0.1 | 1.1 ± 0.3 | 1.2 ± 0.1 |
| Heart | 0.58 ± 0.07 | 0.69 ± 0.10 | 0.52 ± 0.08 | 0.54 ± 0.10 |
| Lungs | 0.83 ± 0.09 | 1.2 ± 0.1 | 0.93 ± 0.22 | 1.1 ± 0.1 |
| Spleen | 0.27 ± 0.02 | 0.38 ± 0.04 | 0.25 ± 0.07 | 0.26 ± 0.01 |
| Kidneys | 1.8 ± 0.3 | 2.0 ± 0.2 | 6.2 ± 0.7 | 3.7 ± 0.5 |
| Stomach | 0.39 ± 0.06 | 0.54 ± 0.02 | 0.32 ± 0.08 | 0.40 ± 0.08 |
| Pancreas | 0.91 ± 0.10 | 1.4 ± 0.4 | 0.39 ± 0.11 | 0.61 ± 0.07 |
| Intestine | 0.57 ± 0.10 | 1.5 ± 0.4 | 0.68 ± 0.09 | 0.54 ± 0.06 |
| Liver | 0.41 ± 0.06 | 1.7 ± 0.3 | 0.38 ± 0.09 | 0.34 ± 0.03 |
| Muscle | 1.1 ± 0.1 | 1.4 ± 0.4 | 0.44 ± 0.15 | 0.73 ± 0.11 |
| Bone | 4.7 ± 0.6 | 5.6 ± 0.9 | 1.4 ± 0.4 | 2.3 ± 0.1 |
| Brain | 0.05 ± 0.01 | 0.06 ± 0.01 | <0.05 | 0.05 ± 0.01 |
| Salivary glands | 1.7 ± 0.2 | 2.5 ± 0.8 | 0.65 ± 0.14 | 1.1 ± 0.1 |
| Gall bladder | 1.1 ± 0.3 | 14 ± 5 | 3.8 ± 0.2 | 2.2 ± 1.0 |
| HT1080-FAP tumor | 8.8 ± 2.9 | 12 ± 4 | 20 ± 3 | 14 ± 2 |
| HT1080 tumor | 2.2 ± 0.5 | 2.1 ± 1.0 | 1.4 ± 0.5 | 1.4 ± 0.4 |
|  |  |  |  |  |
|  | [^177^Lu]Lu-  FAPI-46 | [^177^Lu]Lu-OncoFAP | [^177^Lu]Lu-FAP-2286 | [^177^Lu]Lu-3BP-3940 |
| **Uptake ratios** | n = 5 | n = 4 | n = 3 | n = 3 |
| HT1080-FAP tumor-to-HT1080 tumor ratio | 4.1 ± 1.5 | 6.9 ± 3.2 | 15 ± 4 | 11 ± 4 |
| HT1080-FAP tumor-to-blood ratio | 8.2 ± 3.1 | 9.3 ± 3.9 | 19 ± 5 | 12 ± 2 |
| HT1080-FAP tumor-to-kidneys ratio | 5.1 ± 1.6 | 6.0 ± 1.8 | 3.3 ± 0.6 | 3.6 ± 0.4 |
| HT1080-FAP tumor-to-bone ratio | 1.9 ± 0.6 | 2.3 ± 1.2 | 16 ± 7 | 5.9 ± 0.6 |

**Table S10** Biodistribution data and HT1080-FAP tumor-to-tissue uptake ratios of accumulated activity obtained in HT1080-FAP and HT1080 tumor xenograft-bearing mice at 4 h after injection of the FAP-targeting radioconjugates labeled with lutetium-177. Decay-corrected data of accumulated activity are shown as % IA/g tissue, representing the average ± SD of n = 3–5 mice

|  | **Biodistribution of ^177^Lu-labeled FAPIs at 4 h p.i.**^†^ | | | |
| --- | --- | --- | --- | --- |
|  | [^177^Lu]Lu-  FAPI-46 | [^177^Lu]Lu-OncoFAP | [^177^Lu]Lu-  FAP-2286 | [^177^Lu]Lu-  3BP-3940 |
| **Organ** | n = 3 | n = 4 | n = 3 | n = 5 |
|  | (% IA/g) | (% IA/g) | (% IA/g) | (% IA/g) |
| Blood | 0.59 ± 0.03 | 0.59 ± 0.05 | <0.05 | 0.17 ± 0.08 |
| Heart | 0.32 ± 0.05 | 0.33 ± 0.05 | <0.05 | 0.08 ± 0.04 |
| Lungs | 0.44 ± 0.01 | 0.461 ± 0.004 | 0.08 ± 0.02 | 0.19 ± 0.06 |
| Spleen | 0.24 ± 0.01 | 0.30 ± 0.03 | <0.05 | 0.05 ± 0.02 |
| Kidneys | 1.4 ± 0.2 | 1.4 ± 0.1 | 4.5 ± 0.7 | 0.89 ± 0.32 |
| Stomach | 0.31 ± 0.09 | 0.24 ± 0.01 | <0.05 | 0.07 ± 0.02 |
| Pancreas | 0.67 ± 0.19 | 0.64 ± 0.14 | <0.05 | 0.11 ± 0.04 |
| Intestine | 0.38 ± 0.17 | 0.60 ± 0.20 | 0.08 ± 0.04 | 0.11 ± 0.04 |
| Liver | 0.40 ± 0.04 | 0.53 ± 0.05 | 0.10 ± 0.02 | 0.13 ± 0.03 |
| Muscle | 0.74 ± 0.03 | 0.67 ± 0.18 | <0.05 | 0.17 ± 0.07 |
| Bone | 3.4 ± 0.5 | 2.7 ± 0.3 | 0.12 ± 0.01 | 0.65 ± 0.17 |
| Brain | <0.05 | <0.05 | <0.05 | <0.05 |
| Salivary glands | 1.0 ± 0.1 | 1.1 ± 0.2 | 0.06 ± 0.02 | 0.21 ± 0.08 |
| Gall bladder | 0.77 ± 0.29 | 12 ± 3 | 1.1 ± 0.2 | 1.7 ± 0.7 |
| HT1080-FAP tumor | 8.0 ± 1.3 | 7.6 ± 1.3 | 7.4 ± 1.0 | 11 ± 2 |
| HT1080 tumor | 2.5 ± 1.5 | 1.1 ± 0.1^#^ | 0.26 ± 0.06 | 0.53 ± 0.29 |
|  |  |  |  |  |
|  | [^177^Lu]Lu-  FAPI-46 | [^177^Lu]Lu-OncoFAP | [^177^Lu]Lu-  FAP-2286 | [^177^Lu]Lu-  3BP-3940 |
| **Uptake ratios** | n = 3 | n = 4 | n = 3 | n = 5 |
| HT1080-FAP tumor-to-  HT1080 tumor ratio | 4.0 ± 2.2 | 7.6 ± 2.2^#^ | 30 ± 11 | 24 ± 9 |
| HT1080-FAP tumor-to-blood ratio | 14 ± 2 | 13 ± 2 | 269 ± 96 | 70 ± 18 |
| HT1080-FAP tumor-to-kidneys ratio | 5.8 ± 0.7 | 5.4 ± 0.8 | 1.7 ± 0.4 | 13 ± 4 |
| HT1080-FAP tumor-to-bone ratio | 2.4 ± 0.4 | 2.8 ± 0.4 | 61 ± 12 | 17 ± 2 |

**^†^**Data similar to those reported in Tables S5-S8. ^#^One mouse did not develop HT1080 tumor; hence, n = 3 was considered for the analysis

**Table S11** Biodistribution data and HT1080-FAP tumor-to-tissue uptake ratios of accumulated activity obtained in HT1080-FAP and HT1080 tumor xenograft-bearing mice at 24 h after injection of the FAP-targeting radioconjugates labeled with lutetium-177. Decay-corrected data of accumulated activity are shown as % IA/g tissue, representing the average ± SD of n = 3 mice

|  | **Biodistribution of ^177^Lu-labeled FAPIs at 24 h p.i.** | | | |
| --- | --- | --- | --- | --- |
|  | [^177^Lu]Lu-  FAPI-46 | [^177^Lu]Lu-OncoFAP | [^177^Lu]Lu-  FAP-2286 | [^177^Lu]Lu-  3BP-3940 |
| **Organ** | n = 3 | n = 3 | n = 3 | n = 3 |
|  | (% IA/g) | (% IA/g) | (% IA/g) | (% IA/g) |
| Blood | <0.05 | <0.05 | <0.05 | <0.05 |
| Heart | 0.05 ± 0.01 | 0.058 ± 0.002 | <0.05 | <0.05 |
| Lungs | 0.06 ± 0.01 | 0.058 ± 0.004 | <0.05 | <0.05 |
| Spleen | 0.19 ± 0.04 | 0.11 ± 0.01 | <0.05 | <0.05 |
| Kidneys | 0.60 ± 0.10 | 0.37 ± 0.04 | 4.6 ± 0.7 | 0.105 ± 0.004 |
| Stomach | 0.10 ± 0.08 | 0.08 ± 0.03 | 0.11 ± 0.14 | <0.05 |
| Pancreas | 0.08 ± 0.03 | 0.09 ± 0.01 | <0.05 | <0.05 |
| Intestine | 0.06 ± 0.02 | 0.11 ± 0.05 | 0.11 ± 0.15 | 0.05 ± 0.02 |
| Liver | 0.28 ± 0.04 | 0.38 ± 0.04 | 0.09 ± 0.01 | <0.05 |
| Muscle | 0.12 ± 0.06 | 0.10 ± 0.01 | <0.05 | <0.05 |
| Bone | 0.71 ± 0.18 | 0.52 ± 0.03 | 0.09 ± 0.01 | <0.05 |
| Brain | <0.05 | <0.05 | <0.05 | <0.05 |
| Salivary glands | 0.16 ± 0.07 | 0.19 ± 0.01 | <0.05 | <0.05 |
| Gall bladder | 0.09 ± 0.04 | 0.12 ± 0.05 | 0.06 ± 0.02 | 0.11 ± 0.08 |
| HT1080-FAP tumor | 2.6 ± 0.4 | 1.2 ± 0.1 | 2.6 ± 0.4 | 6.4 ± 0.6 |
| HT1080 tumor | 0.45 ± 0.07 | 0.56 ± 0.51 | 0.14 ± 0.02 | <0.05 |
|  |  |  |  |  |
|  | [^177^Lu]Lu-FAPI-46 | [^177^Lu]Lu-OncoFAP | [^177^Lu]Lu-FAP-2286 | [^177^Lu]Lu-3BP-3940 |
| **Uptake ratios** | n = 3 | n = 3 | n = 3 | n = 3 |
| HT1080-FAP tumor-to-  HT1080 tumor ratio | 6.0 ± 1.7 | 3.4 ± 2.2 | 19 ± 2 | 297 ± 72* |
| HT1080-FAP tumor-to-blood ratio | 81 ± 26 | 36 ± 5 | 1083 ± 241 | 1356 ± 113 |
| HT1080-FAP tumor-to-kidneys ratio | 4.4 ± 1.4 | 3.3 ± 0.5 | 0.56 ± 0.09 | 61 ± 5 |
| HT1080-FAP tumor-to-bone ratio | 3.8 ± 0.9 | 2.3 ± 0.1 | 31 ± 9 | 139 ± 10 |

**References**

1.	Loktev A, Lindner T, Burger EM, Altmann A, Giesel F, Kratochwil C, et al. Development of fibroblast activation protein-targeted radiotracers with improved tumor retention. J Nucl Med. 2019;60:1421-9. doi:10.2967/jnumed.118.224469.

2.	Millul J, Bassi G, Mock J, Elsayed A, Pellegrino C, Zana A, et al. An ultra-high-affinity small organic ligand of fibroblast activation protein for tumor-targeting applications. Proc Natl Acad Sci U S A. 2021;118. doi:10.1073/pnas.2101852118.

3.	Zboralski D, Hoehne A, Bredenbeck A, Schumann A, Nguyen M, Schneider E, et al. Preclinical evaluation of FAP-2286 for fibroblast activation protein targeted radionuclide imaging and therapy. Eur J Nucl Med Mol Imaging. 2022;49:3651-67. doi:10.1007/s00259-022-05842-5.

4.	van der Heide CD, Ntihabose CM, Konijnenberg M, Ma H, Stuurman D, de Ridder C, et al. Head-to-head comparison of [^177^Lu]Lu-FAP-2286 and [^161^Tb]Tb-FAP-2286 efficacy in a PDAC mouse model. EJNMMI Res. 2026. doi:10.1186/s13550-026-01372-5.

5.	Greifenstein L, Gunkel A, Hoehne A, Osterkamp F, Smerling C, Landvogt C, et al. 3BP-3940, a highly potent FAP-targeting peptide for theranostics - production, validation and first in human experience with Ga-68 and Lu-177. iScience. 2023;26:108541. doi:10.1016/j.isci.2023.108541.

6.	Zboralski D, Osterkamp F, von Hacht JL, Bredenbeck A, Hohne A, Libera-Korner J, et al. Preclinical development of 3BP-3940, a fibroblast activation protein-targeted cyclic peptide-based radiotheranostic for imaging and therapy. J Nucl Med. 2026. doi:10.2967/jnumed.125.271452.

7.	Gracheva N, Müller C, Talip Z, Heinitz S, Köster U, Zeevaart JR, et al. Production and characterization of no-carrier-added ^161^Tb as an alternative to the clinically-applied ^177^Lu for radionuclide therapy. EJNMMI Radiopharm Chem. 2019;4:12. doi:10.1186/s41181-019-0063-6.

8.	Mansi R, Wang X, Forrer F, Kneifel S, Tamma ML, Waser B, et al. Evaluation of a 1,4,7,10-tetraazacyclododecane-1,4,7,10-tetraacetic acid-conjugated bombesin-based radioantagonist for the labeling with single-photon emission computed tomography, positron emission tomography, and therapeutic radionuclides. Clin Cancer Res. 2009;15:5240-9. doi:10.1158/1078-0432.CCR-08-3145.

9.	Deberle LM, Tschan VJ, Borgna F, Sozzi-Guo F, Bernhardt P, Schibli R, et al. Albumin-binding PSMA radioligands: impact of minimal structural changes on the tissue distribution profile. Molecules. 2020;25. doi:10.3390/molecules25112542.

10.	Busslinger SD, Becker AE, Vaccarin C, Deberle LM, Renz ML, Groehn V, et al. Investigations using albumin binders to modify the tissue distribution profile of radiopharmaceuticals exemplified with folate radioconjugates. Cancers (Basel). 2023;15. doi:10.3390/cancers15174259.

11.	Borgna F, Barritt P, Grundler PV, Talip Z, Cohrs S, Zeevaart JR, et al. Simultaneous visualization of ^161^Tb- and ^177^Lu-labeled somatostatin analogues using dual-isotope SPECT imaging. Pharmaceutics. 2021;13. doi:10.3390/pharmaceutics13040536.
